# The Association Between Antidiabetic Agents and Clinical Outcomes of COVID-19 Patients With Diabetes: A Bayesian Network Meta-Analysis

**DOI:** 10.3389/fendo.2022.895458

**Published:** 2022-05-27

**Authors:** Yidan Chen, Xingfei Lv, Sang Lin, Mohammad Arshad, Mengjun Dai

**Affiliations:** ^1^Department of Rheumatology & Immunology, West China Hospital of Sichuan University, Chengdu, China; ^2^Department of Orthopedics, People’s Hospital of Zhongjiang County, Deyang, China; ^3^Department of Rheumatology& Immunology, China-Japan Friendship Hospital, Beijing, China; ^4^Department of Pediatrics Surgery, Lok Nayak Hospital, New Delhi, India; ^5^Department of Interventional Oncology, Renji Hospital, School of Medicine, Shanghai Jiao Tong University, Shanghai, China

**Keywords:** diabetes, antidiabetic agents, COVID-19, mortality, Bayesian network meta-analysis

## Abstract

**Aims:**

This study aimed to assess the impact of different antidiabetic agents on individuals with diabetes and COVID-19.

**Methods:**

We searched PubMed, Web of Science, Embase, and Cochrane Library databases from inception to October 31, 2021 and included seven antidiabetic agents. The data were pooled *via* traditional pairwise meta-analysis and Bayesian network meta-analysis.

**Results:**

The pairwise meta-analysis included 35 studies. Metformin (odds ratio (OR), 0.74; P=0.001), dipeptidyl peptidase-4 inhibitors (DPP4i) (OR, 0.88; P=0.04), sodium-glucose cotransporter-2 inhibitors (SGLT2i) (OR, 0.82; P=0.001), and glucagon-like peptide-1 receptor agonists (GLP1RA) (OR, 0.91; P=0.02) treatment were associated with lower COVID-19 mortality in individuals with diabetes compared to respective non-users. However, insulin treatment resulted in higher mortality (OR, 1.8; P=0.001). Mortality did not significantly differ in sulfonylurea (OR, 0.97; P=0.56) and thiazolidinediones (TZDs) (OR, 1.00; P=0.96) users. Furthermore, due to limited data, we analyzed five antidiabetic agents (metformin, DPP4i, sulfonylurea, insulin, and SGLT2i) and found no association between them and severe disease risk (all P>0.05). The Bayesian network meta-analysis included 18 studies. GLP1RA and SGLT2i had the highest first and second rank probability (67.3% and 62.5%, respectively). Insulin showed the maximum probability of ranking seventh (97.0%). Metformin had the third and fourth highest rank probability of 44.8% and 38.9%, respectively. Meanwhile, DPP4i had the fifth-highest rank probability of 42.4%, followed by sulfonylurea at 45.1%.

**Conclusion:**

Metformin, DPP4i, SGLT2i, and GLP1RA treatments were highly possible to reduced COVID-19 mortality risk in individuals with diabetes, while insulin might be related to increased mortality risk. Sulfonylurea and TZDs treatments were not associated with mortality. None of the antidiabetic agents studied were associated with the risk of severe disease. Additionally, GLP1RA probably had the most significant protective effect against death, followed by SGLT2i and metformin.

**Systematic Review Registration:**

PROSPERO (CRD42021288200)

## Introduction

The ongoing pandemic of Coronavirus disease-19 (COVID-19) is an emerging, rapidly evolving situation that poses challenges for diabetic patients. Previous studies have demonstrated that diabetes is an independent risk factor for worse outcomes and in-hospital mortality in COVID-19 patients [1]. The percentage of COVID-19 patients with diabetes in hospitalization was high, with adverse effects on their prognosis, and most COVID-19 fatalities occurred among diabetic patients. It is necessary to decide a relatively beneficial treatment plan for diabetic patients with COVID-19 among the massive diabetes treatment plans.

Antidiabetic agents have been demonstrated to exhibit an antidiabetic role and anti-inflammatory and immunomodulatory effects (1, 2). Numerous studies have been conducted to determine the impact of antidiabetic agents on clinical outcomes of diabetic patients with COVID-19. Additionally, some pairwise meta-analyses researched the role of a specific antidiabetic agent on the mortality and severe poor outcomes of COVID-19, including metformin, dipeptidyl peptidase 4 inhibitors (DPP4i), sulfonylurea, insulin, and glucagon-like peptide-1 receptor agonists (GLP1RA) (1, 3, 4). However, these investigations merely compared the outcomes of users with specific antidiabetic agents to those of non-users. The mortality risks between different antidiabetic agents in these patients remain unclear.

To date, no comprehensive network meta-analysis has been performed to explore the associations between different antidiabetic drugs and COVID-19 patients’ outcomes. The current analysis aims to evaluate the association between different antidiabetic agents with the risk of COVID-19 mortality, as well as the risk of severe disease outcomes among individuals with diabetes.

## Material and Methods

### Literature Search

The literatures of any design were searched in PubMed, Embase, and Cochrane Library databases from inception to October 30, 2021, following the Meta-analyses Of Observational Studies in Epidemiology (MOOSE) guidelines as described (5). The PROSPERO registration number is CRD42021288200. The search terms included antidiabetic agents, metformin, DPP4i, sulfonylurea, thiazolidinediones (TZDs), insulin, sodium-glucose cotransporter-2 inhibitors (SGLT2i), GLP1RA, glinides, α-glycosidase inhibitors, and COVID-19. Each search strategy included a combination of one antidiabetic agent and COVID-19. The exact search terms are shown in **Supplementary Table 1** (**S Table 1**). We limited our search by the English language. The retrieved articles were imported into EndnoteX9 and extracted by screening their titles and abstracts. Duplicate studies and multiple reports using the same data were removed. After preliminary screening, nonconforming literature was excluded by reading the full text, and the final remaining articles were included in this study.

### Inclusion and Exclusion Criteria

This study comprised studies that met the following criteria: (1) original studies evaluating the clinical outcomes of diabetic COVID-19 patients receiving specific antidiabetic agents at home or in the hospital; (2) study designs including randomized controlled trials (RCT), and observational studies; (3) studies published in English. The exclusion criteria included the following: (1) reviews, systematic reviews, correspondences, or *in vitro* animal studies; (2) studies with incomplete data and indeterminate outcome endpoints; (3) abstracts, unpublished studies, and retracted manuscripts.

### Data Extraction and Quality Assessment

Two independent reviewers used a predetermined data collection table to extract relevant data. Disagreements were solved by discussion and following a third author’s opinion. The main extracted data included the following: study characteristics (first author, publication date, country, and study design), patient characteristics (sample size, mean age, and male proportion), as well as study’s outcomes data. Newcastle-Ottawa Quality Assessment Scale criteria were utilized to assess the risk of bias for the included studies (6, 7). According to this scale, a study can be awarded a maximum of nine stars; four stars for study selection, two stars for comparability, and three stars for outcome sections.

### Outcomes of Interest

Our primary outcome was the mortality of individuals with diabetes and COVID-19. The secondary outcome was the incidence of severe disease. Here, we defined the cases with poor outcomes as those requiring intensive care unit admission, mechanical ventilation of acute respiratory distress syndrome, disseminated intravascular coagulation, or others described in the individual study as severe disease.

### Statistical Analysis

Meta-analysis and Bayesian network were conducted only in studies >3 per outcome. Meta-analysis was performed using Stata 16.0 (Stata Corp) software. Both chi-squared and I^2^ tests estimated heterogeneity between studies. The pooled effect sizes were analyzed using a random-effects model (I^2^ >0.5, P <0.1) and fixed-effects model (I^2^ <0.5, P >0.1). As reported by individual cohort studies, pooled OR was estimated by combining relative risk (RR) or odds ratio (OR) (hazards ratio was presumably equivalent to RR). If studies included unadjusted and covariate-adjusted data, we selected the latter. To stabilize the variances, we transformed each study’s effect estimates and their confidence intervals (CI) to natural logarithms (8, 9). If the number of studies was >5, publication bias was evaluated using Begg’s test, Egger’s test, and the trim-and-fill method. Subgroup analyses based on data types (adjusted or unadjusted data), effect size types (OR or RR), diabetes types (only T2DM or not only T2DM), and medication use timing (use before admission or in-hospital) would be established when the number of studies in a subgroup was greater than three. Meta-regression analysis was performed to assess the potential impact of the study’s sample size, patients’ mean age, and gender on the pooled results.

The Bayesian network meta-analysis was performed using R 4.1 software with gemtc and rjags packages as previously described (10). The network meta-analysis employed OR and 95% credibility intervals (95% CrIs). Our study’s consistency was determined by comparing the fit of consistency and inconsistency models using deviance information criteria (differences < 5 are considered consistency fitting) (11). We calculated the rank probabilities of the effects of all drugs available in the network. Additionally, the probability of the first to seventh of each drug was visualized using radar plots of ranking probability for the network. P ≤ 0.05 was considered statistically significant.

## Results

### Study Selection and Characteristics

**Figure 1:**
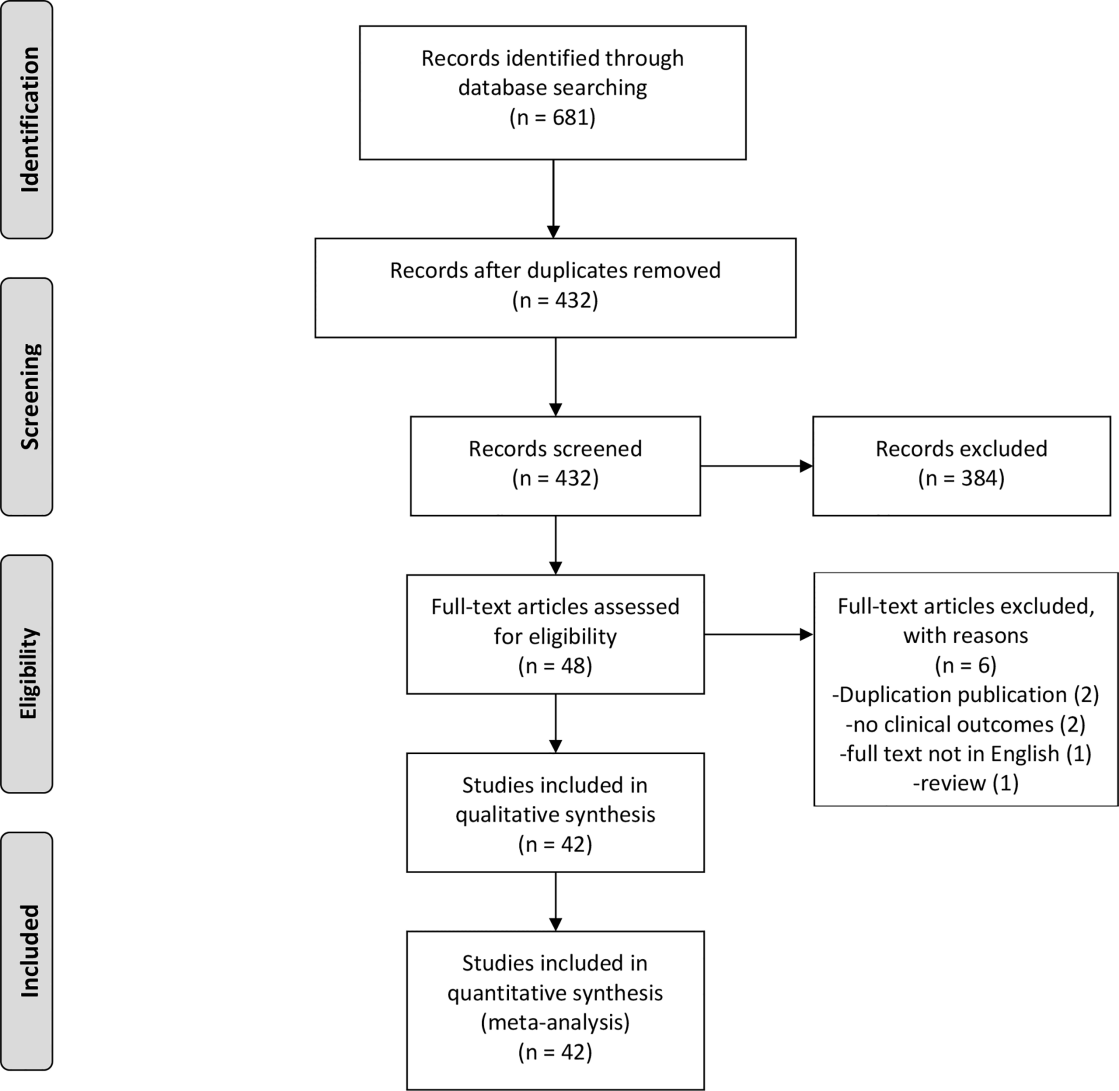
The flow diagram of study selection.

The search strategy identified 681 potentially relevant studies, of which 249 were excluded due to duplication. Among the remaining 432 studies, 384 were excluded due to their titles or abstracts. Therefore, 48 studies were eligible for full-text review. After re-excluding six articles, this study comprised 42 full-text articles (**Figure 1**). The vast majority of them were observational studies (6 cross-sectional studies, 1 case-control study, and 34 cohort studies) and only one article was RCT. **Table 1** summarizes the baseline characteristics of patients in the included studies.

**Table 1:** Clinical characteristics of the studies included in the review.

Author (year)	Country	Included in	Type of study	Sample	Sample characteristics	Results
Silverii, G. A (2021) (12)	Italy	Bayesian network meta-analysis and pairwise meta-analysis	cross-sectional study	Patients with COVID-19 and DM; Sample size: 159	Mean age: 73.31; Male: 54.1%	No medication for diabetes was associated with differences in risk of COVID-19 fatality, with the only exception of metformin (RR 0.6; 95%CI 0.39-0.93).
Kahkoska, A. R (2021) (13)	USA	Bayesian network meta-analysis	retrospective cohort study	Patients with COVID-19 and DM; Sample size: 12446	Mean age: 58.6; Male: 46.6%; White: 62.5%	Both GLP1-RA and SGLT2i use were associated with lower 60-day mortality compared with DPP4i use (OR 0.54 [95% CI 0.37–0.80] and 0.66 [0.50–0.86], respectively).
Ramos-Rincón, J. M (2021) (14)	Spain	Bayesian network meta-analysis and pairwise meta-analysis	cross-sectional study	Patients with COVID-19 and T2DM and age≥80; Sample size: 790	Mean age: 85; Male: 52.9%;	The preadmission cardiometabolic medications found to be independent protective factors against in-hospital mortality were the use of DPP-4i (adjusted OR 0.502, 95% CI: 0.309–0.815, p = 0.005).
Orioli, L (2021) (15)	Belgium	Bayesian network meta-analysis	retrospective cohort study	Patients with COVID-19 and DM; Sample size: 73	Mean age: 69; Male: 48%; White: 79.5%	Death cases were less often treated with metformin (P<0.036)
Israelsen, S. B (2021) (16)	Denmark	Bayesian network meta-analysis	retrospective cohort study	Patients with COVID-19 and DM; Sample size: 1970	/	Current users of GLP‐1 RAs had an adjusted RR of 0.89 (95%CI 0.34‐2.33), while users of DPP‐4i had an adjusted RR of 2.42 (95%CI 0.99‐5.89) for 30‐day mortality compared with SGLT‐2i use.
Sourij, H (2021) (17)	Austria	Bayesian network meta-analysis and pairwise meta-analysis	combined prospective and retrospective cohort study	Patients with COVID-19 and DM; Sample size: 238	Mean age: 71.1; Male: 63.6%;	With regard to medication use, no difference was observed for any other glucose‐lowering medication between people who survived or died.
Nyland, J. E (2021) (18)	USA	Bayesian network meta-analysis and pairwise meta-analysis	retrospective cohort study	Patients with COVID-19 and T2DM; Sample size: 29516	Mean age: 60.9; Male: 48.2%; White: 47.9%	Use of glucose-regulating medications such as GLP-1R agonists, DPP-4 inhibitors, or pioglitazone may improve outcomes for COVID-19 patients with T2DM.
Cheng, X (2021) (19)	China	Bayesian network meta-analysis	retrospective cohort study	Patients with COVID-19 and T2DM; Sample size: 50	Mean age: 56.0; Male: 54%; White: 0%	In-hospital insulin usage attempted to increase the invasive ventilation (34.8% vs. 3.7%, adjust P = 0.043), independent of age and blood glucose.
Elibol, A (2021) (20)	Turkey	Bayesian network meta-analysis	cross-sectional study	Patients with COVID-19 and T2DM; Sample size: 432	Mean age: 63.3; Male: 45.6%	No oral anti-diabetics was found to be associated with COVID-19 related death.
Luk, A. O. Y (2021) (21)	China	Bayesian network meta-analysis and pairwise meta-analysis	retrospective cohort study	Patients with COVID-19 and T2DM; Sample size: 1220	Mean age: 65.3; Male: 54.3%	Users of metformin and DPP-4 inhibitors had fewer adverse outcomes from COVID-19 compared with non-users, whereas insulin and sulphonylurea might predict a worse prognosis.
Li, J (2021) (22)	China	Bayesian network meta-analysis	retrospective cohort study	Patients with COVID-19 and T2DM; Sample size: 131	Mean age: 66.8; Male: 56.5%; White: 0%	Using metformin (p = .02) and acarbose (p = 0.04), alone or both together (p = 0.03), after admission were significantly more likely to survive than those who did not use either metformin or acarbose.
Lally, M. A (2021) (23)	USA	Bayesian network meta-analysis	retrospective cohort study	Patients with COVID-19 and DM; Sample size: 775	Mean age: 75.6; Male: 97.3%; White: 66.3%	Comparing to those not receiving diabetes medications, residents taking metformin were at significantly reduced hazard of death (adjusted HR 0.48, 95%CI 0.28, 0.84) over the subsequent 30 days from COVID-19 diagnosis. There was no association with insulin (adjusted HR 0.99, 95% CI 0.60, 1.64) or other diabetes medications (adjusted HR 0.71, 95% CI 0.38, 1.32).
Crouse, A. B (2020) (24)	USA	Bayesian network meta-analysis and pairwise meta-analysis	retrospective cohort study	Patients with COVID-19 and DM; Sample size: 239	Mean age:/; Male: 50.6%; White: 27.6%	Metformin may provide a protective approach in high-risk population.
Pérez-Belmonte, L. M (2020) (25)	Spain	Bayesian network meta-analysis and pairwise meta-analysis	retrospective cohort study	Patients with COVID-19 and T2DM; Sample size: 2666	Mean age: 74.9; Male: 61.9%	In patients with type 2 diabetes mellitus admitted for COVID-19, at-home glucose-lowering drugs showed no significant association with mortality and adverse outcomes.
Wargny, M (2021) (26)	France	Bayesian network meta-analysis and pairwise meta-analysis	retrospective cohort study	Patients with COVID-19 and DM; Sample size: 2796	Mean age:/; Male: 63.7%; White: 58.1%	Insulin was associated with a greater risk of death while routine metformin therapy was negatively associated with death.
Khunti, K (2021) (27)	UK	Bayesian network meta-analysis and pairwise meta-analysis	retrospective cohort study	Patients with T2DM; Sample size: 2851465	Mean age:/; Male: 55.9%; White: 66.1%	The adjusted HR associated with recorded versus no recorded prescription was 0.77 (95% CI 0.73–0.81) for metformin and 1.42 (1.35–1.49) for insulin.
Solerte, S. B (2020) (28)	Italy	Bayesian network meta-analysis and pairwise meta-analysis	retrospective case-control study	Patients with T2DM; Sample size: 338	Mean age: 69; Male: 70.4%;	Treatment with sitagliptin at the time of hospitalization was associated with reduced mortality (18% vs. 37% of deceased patients; hazard ratio 0.44 [95% CI 0.29–0.66]; P = 0.0001).
Meijer, R. I (2021) (29)	Netherlands	Bayesian network meta-analysis and pairwise meta-analysis	prospective cohort study	Patients with COVID-19 and T2DM; Sample size: 565	Mean age: 69.4; Male: 64.1%;	Outpatient use of a DPP-4 inhibitor does not affect the clinical outcomes of patients with type 2 diabetes who are hospitalized because of COVID-19 infection.
Cheng X (2020) (30)	China	Pairwise meta-analysis	retrospective cohort study	Patients with COVID-19 and T2DM; Sample size: 1213	Mean age: 63; Male: 52.1%;	Metformin use was significantly associated with a higher incidence of acidosis, particularly in cases with severe COVID-19, but not with 28-day COVID-19-related mortality.
Luo P (2020) (31)	China	Pairwise meta-analysis	retrospective cohort study	Patients with COVID-19 and DM; Sample size: 283	Mean age: 64; Male: 55.1%;	Antidiabetic treatment with metformin was associated with decreased mortality compared with diabetics not receiving metformin.
Bramante CT (2021) (32)	USA	Pairwise meta-analysis	retrospective cohort study	Patients with COVID-19 and DM; Sample size: 9555	Mean age:/; Male: 47.3%; White: 69.2%	Metformin was associated with a decrease in mortality from COVID‐19, in the propensity‐matched cohorts, OR 0.38 (0.16, 0.91; p = 0.03).
Ghany R (2021) (33)	USA	Pairwise meta-analysis	retrospective cohort study	Patients with COVID-19; Sample size: 1139	Mean age: 71.1; Male: 40.3%	The relative hazard (RH) of death for metformin users was 0.34; 95% CI 0.19–0.59. The RH of ARDS for metformin users was 0.32; 95% CI 0.22–0.45.
Jiang N (2021) (34)	China	Pairwise meta-analysis	retrospective cohort study	Patients with COVID-19 and T2DM; Sample size: 148	Mean age: 65; Male: 47.3%	In the mixed-effected model, metformin use was associated with the lower incidence of ARDS, but no significant association with 30-day all-cause mortality.
Lalau J D (2021) (35)	France	Pairwise meta-analysis	retrospective cohort study	Patients with COVID-19 and T2DM; Sample size: 2449	Mean age: 70.9; Male: 64.0%	The odds ratios for primary outcome and death (OR [95%CI], metformin users vs non-users) were 0.783 [0.615−0.996] and 0.710 [0.537−0.938] on day 28, respectively.
Oh T K (2021) (36)	South Korea	Pairwise meta-analysis	retrospective cohort study	Patients with COVID-19 and T2DM; Sample size: 11892	Male: 44.9%	Metformin use was not associated with hospital mortality (OR: 1.26, 95% CI: 0.81–1.95; P = 0.301).
Bramante CT2 (2021) (37)	USA	Pairwise meta-analysis	retrospective cohort study	Patients with COVID-19 and T2DM; Sample size: 6256	Mean age: 73; Male: 47.2%	In unadjusted analyses, metformin was associated with decreased mortality. But the association was not statistically significant in the adjusted analysis.
Chen Y (2020) (38)	China	Pairwise meta-analysis	retrospective cohort study	Patients with COVID-19 and DM; Sample size: 120	Mean age: 64	None of the glucose-lowering medications (metformin, insulin, α-glycosidase, secretagogues, or DPP-4 inhibitors) were associated with in-hospital death.
Do JY (2020) (39)	South Korea	Pairwise meta-analysis	retrospective cohort study	Patients with COVID-19 and T2DM; Sample size: 1865	Mean age: 61; Male: 58.7%	No definite association could be found between metformin use and clinical outcomes, including survival
Gao Y (2020) (40)	China	Pairwise meta-analysis	retrospective cohort study	Patients with COVID-19 and T2DM; Sample size: 110	Mean age: 65; Male: 41.8%	Antidiabetic therapy with metformin was associated with a higher risk of severe illness (adjusted odds ratio 3.964, 95% confidence interval 1.034–15.194).
Kim MK (2020) (41)	South Korea	Pairwise meta-analysis	cross-sectional study	Patients with COVID-19 and DM; Sample size: 470	Mean age: 69; Male: 42.8%	The use of metformin or insulin tended to be associated with less severe disease and lower mortality, although these findings did not achieve statistical significance
Saygili ES (2021) (42)	Turkey	Pairwise meta-analysis	retrospective cohort study	Patients with COVID-19 and DM; Sample size: 240	Mean age: 69; Male: 52.5%	Preadmission metformin usage is associated with reducing all-cause mortality.
Wander PL (2021) (43)	USA	Pairwise meta-analysis	cross-sectional study	Patients with COVID-19 and DM; Sample size: 64892	White: 66%	Among veterans with diabetes and COVID-19, insulin use were directly associated with adverse outcomes, while use of a GLP1-RA, metformin, and SGLT2i was inversely associated.
Tamura RE (2021) (44)	Brazil	Pairwise meta-analysis	retrospective cohort study	Patients with COVID-19 and DM; Sample size: 188	Male: 62.8%	Patients that used metformin during hospitalization have a better prognosis and reduced risk of death.
Dave JA (2021) (45)	South Africa	Pairwise meta-analysis	retrospective cohort study	Patients with COVID-19 and DM; Sample size: 5708	Mean age: 57; Male: 37.1%	Use of insulin (OR1.49, 95% CI: 1.27; 1.74) was associated with an increased mortality whereas use of metformin (OR 0.77, 95% CI: 0.64; 0.92) was associated with a reduction in mortality.
Cariou B (2020) (46)	France	Pairwise meta-analysis	cross-sectional study	Patients with COVID-19 and DM; Sample size: 1317	Mean age: 69.8; Male: 64.9%	Metformin use was lower in people who died.
Fadini GP (2020) (47)	Italy	Pairwise meta-analysis	retrospective cohort study	Patients with COVID-19 and T2DM; Sample size: 85	Mean age: 70.3; Male: 64.7%	The current study does not support the hypothesis that DPP-4is might be protective against COVID-19.
Rhee SY (2021) (48)	South Korea	Pairwise meta-analysis	retrospective cohort study	Patients with COVID-19 and DM; Sample size: 832 and 704	Male: 53.5% and 40.3%	DPP-4i is significantly associated with a better clinical outcome of patients with COVID-19.
Dalan R (2021) (49)	Singapore	Pairwise meta-analysis	retrospective cohort study	Patients with COVID-19 and DM; Sample size: 76	/	Patients on DPP-4 inhibitors were more likely to require ICU admission.
Noh Y (2021) (50)	South Korea	Pairwise meta-analysis	retrospective cohort study	Patients with COVID-19 and T2DM; Sample size: 586	/	Compared with use of other second- or third-line antidiabetic drugs, use of DPP-4 inhibitors was not associated with adverse COVID-19-related outcomes among patients with T2DM.
Roussel R (2021) (51)	France	Pairwise meta-analysis	retrospective cohort study	Patients with COVID-19 and T2DM; Sample size: 2449	Mean age: 70.9; Male: 64%	No significant association between the use of DPP‐4 inhibitors and the risk of tracheal intubation and death.
Zhou JH (2020) (52)	China	Pairwise meta-analysis	retrospective cohort study	Patients with COVID-19 and T2DM; Sample size: 444	Mean age: 64; Male: 48.6%	The author did not observe any significant difference between patients taking DPP4i drugs and those taking other oral hypoglycemic drugs regarding the incidence or risk of all-cause mortality.
Kosiborod MN (2021) (53)	USA	Pairwise meta-analysis	RCT	Patients with COVID-19 and T2MD; Sample size: 1250	Mean age: 61.4; Male: 42.6%	Dapagliflozin did not significantly reduce the rates of organ dysfunction or death or improve recovery

DM, diabetes mellitus; T2DM, type 2 diabetes mellitus.

### Quality Assessment

Newcastle-Ottawa Quality Assessment measured the quality of studies. The quality scores of studies ranged from seven to nine stars with a median of eight stars (**S Table 2**).

### Meta-analysis of the Impact of Using Antidiabetic Agents on Patients’ Clinical Outcomes

The pairwise meta-analysis included 35 studies (12, 14, 17, 18, 21, 24–53), of which 25 (12, 14, 17, 21, 24–27, 30–46) were related to metformin, 21 (12, 14, 17, 18, 21, 25–29, 36, 38, 41, 43, 46–52) were linked to DPP4i, 10 (12, 17, 21, 26, 27, 36, 41, 43, 46, 49) were associated with sulfonylurea, 5 (12, 18, 27, 36, 43) were related to TZDs, 14 (12, 14, 17, 21, 24–27, 36, 38, 41, 43, 45, 46) were linked to insulin, 8 (12, 14, 17, 27, 41, 43, 49, 53) were correlated with SGLT2i, 7 (12, 14, 18, 26, 27, 43, 46) were linked to GLP1RA, 2 (27, 38) were associated with acarbose (α-glycosidase inhibitors), and one (27) was correlated with glinides. Some of these studies included multiple different medications.

**Figure 2:**
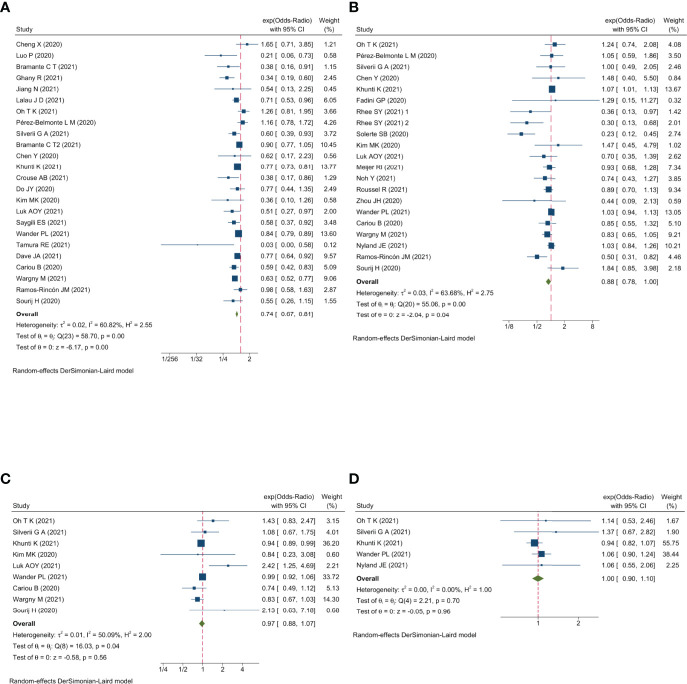
Forest plot showing the association between antidiabetic agents use and mortality. **(A)** Metformin; **(B)** DPP4i; **(C)** Sulfonylurea; **(D)** TZDs.

**Figure 3:**
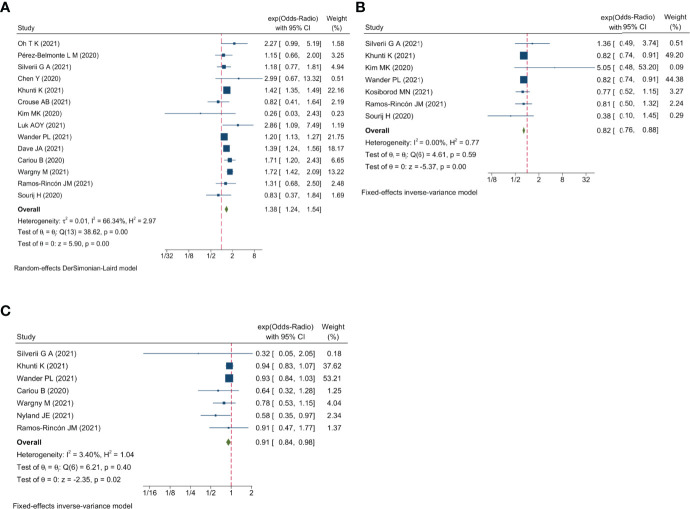
Forest plot showing the association between antidiabetic agents use and mortality. **(A)** Insulin; **(B)** SGLT2i; **(C)** GLP1RA.

**Figure 4:**
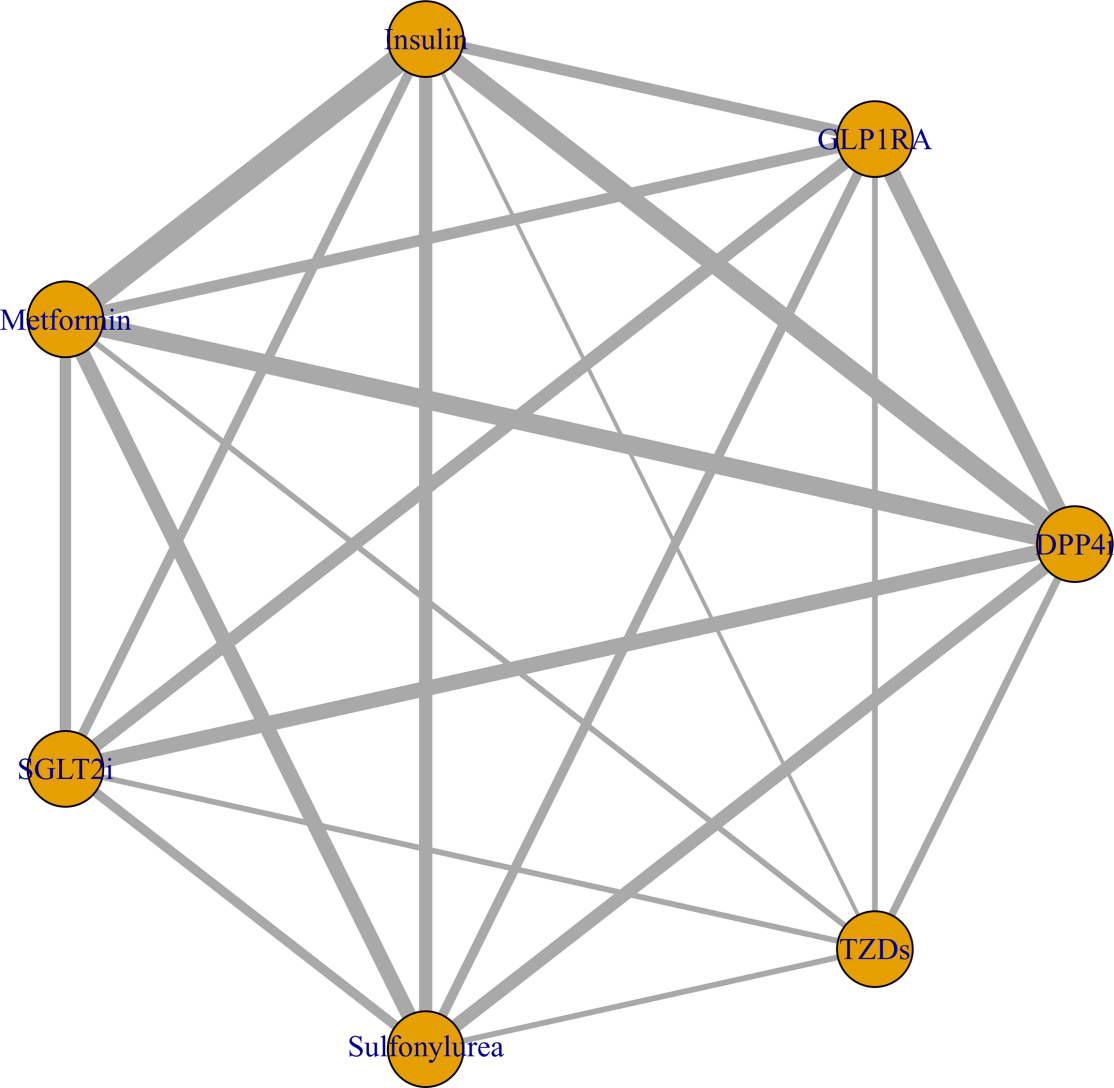
Network plot of the association between different antidiabetic agents and the risk of COVID-19 mortality in diabetic patients. The thickness of lines corresponds to the number of trials.

Qualitative meta-analysis revealed that metformin users had a significantly reduced mortality rate than non-users. (**Figure 2A**; pooled OR, 0.74; 95% CI: 0.67-0.81; P=0.00). Subgroup analyses based on data types, effect size types, and diabetes types respectively revealed lower mortality in metformin users in each subgroup (**S Figures 1A**, **2A** and **3A**, all subgroups P < 0.05). In the Subgroup analyses based on medication use timing, metformin use before admission was associated with a significantly reduced mortality rate (pooled OR, 0.76; 95% CI: 0.69-0.84), but no such an association was observed in in-hospital use subgroup (pooled OR, 0.41; 95% CI: 0.11-1.60) (**S Figures 4A**). Using DPP4i in individuals with diabetes was also associated with reduced mortality from COVID-19 (**Figure 2B**; pooled OR, 0.88; 95% CI: 0.78-1.00; P=0.04). Further subgroup analysis indicated that DPP4i use remained linked to a statistically decreased mortality after adjusting for age, gender, and other characteristics (**S Figure 1B**; pooled OR, 0.86; 95% CI: 0.75-0.98; P=0.02) whereas was not statistically significant in unadjusted data (P > 0.05). However, in subgroup analyses based on effect size types, diabetes types, and DPP4i use timing, there was no statistically significant difference in each subgroup (**S Figures 2B**, **3B**, and **4B**, all subgroups P > 0.05). Thus, caution is needed when interpreting the effect of DPP4i on individuals with diabetes and COVID-19. For sulfonylurea or TZDs medications, no significant difference in mortality was observed between medication users and non-users (sulfonylurea: **Figure 2C**; pooled OR, 0.97; 95% CI: 0.88-1.07; P=0.56, TZDs: **Figure 2D**; pooled OR, 1.00; 95% CI: 0.90-1.10; P=0.96). Subgroup analysis based on data types, effect size types, diabetes types, and sulfonylurea use timing demonstrated similar results respectively (**S Figures 1C**, **2C**, **3C**, and **4C**; all subgroups P > 0.05). Insulin treatment was significantly associated with increased mortality compared to non-users (**Figure 3A**; pooled OR, 1.38; 95% CI: 1.24-1.54; P=0.00). In subgroup analysis based on data types, effect size types and diabetes types, there was also higher mortality in each insulin subgroup (**S Figures 1D**, **2D** and **3D**, all subgroups P < 0.05); while subgroup analysis based on insulin using timing revealed that higher mortality was only observed in before-admission use subgroup (**S Figures 4D**, pooled OR, 1.43; 95% CI: 1.25-1.64). Finally, therapy of SGLT2i or GLP1RA was significantly associated with a reduction in mortality compared to non-users (SGLT2i: **Figure 3B**; pooled OR, 0.82; 95% CI: 0.76-0.88; P=0.00, GLP1RA: **Figure 3C**; pooled OR, 0.91; 95% CI: 0.84-0.98; P=0.02). Subgroup analysis was not performed due to insufficient SGLT-2i or GLP-1RA treatment data. Furthermore, meta-regression analysis disclosed that the study’s sample size, patients’ mean age, and gender did not significantly impact the association of antidiabetic agents on patients’ mortality (all P > 0.05) (**Table 2**).

**Table 2:** The results of publication bias and regression analysis for mortality.

Mortality	Studies trimmed/total studies	OR (95% CI)	P value	Begger’s test	Egger’s test	Trim-and-fill analysisOR (95% CI)	Regression for Sample size	Regression for mean age	Regression for male proportion
Metformin	7/24	0.74 (0.67-0.81)	0.00	0.14	0.00	0.78 (0.71-0.87)	0.68	0.54	0.78
DPP4i	0/21	0.88 (0.78-0.99)	0.04	0.74	0.04	0.88 (0.78-0.99)	0.28	0.30	0.77
Sulfonylurea	2/9	0.97 (0.88-1.07)	0.56	0.25	0.11	0.94 (0.84-1.07)	0.65	0.37	0.10
TZDs	3/5	1.0 (0.90-1.10)	0.96	0.81	0.35	0.99 (0.90-1.09)	0.19	0.61	0.50
Insulin	0/14	1.38 (1.24-1.54)	0.00	0.44	0.92	1.38 (1.24-1.54)	0.87	0.57	0.47
SGLT2i	0/7	0.82 (0.76-0.88)	0.00	1.00	0.58	0.82 (0.76-0.88)	1.00	0.97	0.76
GLP1RA	3/7	0.91 (0.84-0.99)	0.02	0.13	0.04	0.93 (0.86-1.00)	0.52	0.31	0.99

**Figure 5:**
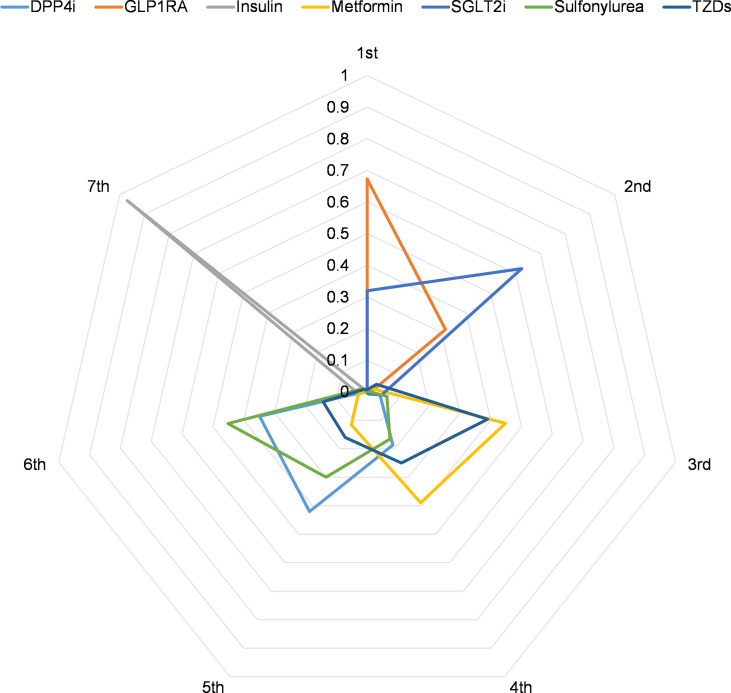
Radar plot of the ranking probability for COVID-19 mortality.

Most studies did not report the outcome of severe disease. Due to a scarcity of data, a qualitative meta-analysis of only five antidiabetic agents was performed (metformin, DPP4i, sulfonylurea, insulin, and SGLT2i). The pooled results demonstrated no significant associations between antidiabetic agent use and risk of severe disease (all P > 0.05) (**S Figure 5**).

Begg’s and Egger’s tests revealed no publication bias for sulfonylurea, TZDs, insulin, and SGLT2i mortality (all P > 0.05), while Egger’s test manifested publication bias for metformin, DPP4i, and GLP1RA (Egger’s test P = 0.00, 0.04, and 0.04, respectively). The trim-and-fill analysis suggested no evidence of a significant difference between adjusted and original values of all antidiabetic agents (**Table 2** and **S Figure 6**), implying that the results in this meta-analysis were relatively robust.

### Bayesian Network Meta-Analysis of the Association Between Antidiabetic Agents and Mortality

The Bayesian network meta-analysis included 18 studies (12–29) and included seven different antidiabetic agents: metformin, DPP4i, sulfonylurea, TZDs, insulin, SGLT2i, and GLP1RA. Due to lack of relevant data, we assessed only the association between different antidiabetic agents and mortality in this Bayesian network meta-analysis and did not examine the risk of severe disease. The network plot is displayed in **Figure 4**.

Using ranking probabilities, Bayesian analysis depicted an overall comparison of the effect of each antidiabetic drug on mortality risk in the network (**Figure 5**). GLP1RA and SGLT2i had the first and second highest rank probabilities of reducing mortality (67.3% and 62.5%, respectively). Insulin had the highest seventh rank probability (97.0%). Metformin had the third and fourth rank probability of 44.8% and 38.9%, respectively. Meanwhile, DPP4i had the fifth-highest rank probability of 42.4%, followed by sulfonylurea at 45.1%.

When taking insulin as a reference, all other antidiabetic agents were associated with a decreased mortality (OR for metformin, 0.56; 95% CrI, 0.42-0.73, OR for DPP4i, 0.66; 95% CrI, 0.49-0.88, OR for sulfonylurea, 0.68; 95% CrI, 0.47-0.97, OR for TZDs, 0.57; 95% CrI, 0.36-0.93, OR for SGLT2i, 0.36; 95% CrI, 0.24-0.55, OR for GLP1RA, 0.33; 95% CrI, 0.23-0.48). Compared with metformin, DPP4i, or sulfonylurea, only GLP1RA and SGLT2i could significantly reduce the mortality, whereas insulin increased the mortality (**Table 3**). All but SGLT2i increased mortality when compared to GLP1RA. Compared with SGLT2i, significantly increased mortality was observed in DPP4i, insulin, metformin, and sulfonylurea treatment groups (**Table 3**).

**Table 3:** The results of Bayesian network analysis.

	Metformin	DPP4i	Sulfonylurea	TZDs	Insulin	SGLT2i	GLP1RA
Metformin	–	0.84 (0.63, 1.1)	0.82 (0.58, 1.2)	0.98 (0.6, 1.6)	**0.56 (0.42, 0.73)**	**1.6 (1.0, 2.3)**	**1.7 (1.2, 2.5)**
DPP4i	1.2 (0.89, 1.6)	–	0.98 (0.69, 1.4)	1.2 (0.73, 1.8)	**0.66 (0.49, 0.88)**	**1.8 (1.2, 2.6)**	**2.0 (1.4, 2.8)**
Sulfonylurea	1.2 (0.85, 1.7)	1.0 (0.72, 1.5)	–	1.2 (0.71, 1.9)	**0.68 (0.47, 0.97)**	**1.9 (1.2, 2.9)**	**2.1 (1.4, 3.1)**
TZDs	1.0 (0.65, 1.7)	0.86 (0.55, 1.4)	0.84 (0.51, 1.4)	–	**0.57 (0.36, 0.93)**	1.6 (0.94, 2.6)	**1.7 (1.1, 2.9)**
Insulin	**1.8 (1.4, 2.4)**	**1.5 (1.1, 2.0)**	**1.5 (1.0, 2.1)**	**1.8 (1.1, 2.8)**	–	**2.8 (1.8, 4.1)**	**3.1 (2.1, 4.4)**
SGLT2i	**0.64 (0.44, 0.98)**	**0.54 (0.38, 0.81)**	**0.53 (0.35, 0.84)**	0.63 (0.38, 1.1)	**0.36 (0.24, 0.55)**	–	1.1 (0.74, 1.7)
GLP1RA	**0.59 (0.41, 0.85)**	**0.49 (0.36, 0.7)**	**0.48 (0.32, 0.74)**	**0.57 (0.35, 0.93)**	**0.33 (0.23, 0.48)**	0.91 (0.59, 1.4)	–

An odds ratio lower than 1 favors the row-defining treatment. Statistically significant results are in boldface.

Consistency results of direct and indirect estimates were detected with relevant P-value larger than 0.05 in any closed loops using the node-splitting method (all P > 0.05, **S Figure 7**). The Brooks-Gelman-Rubin plots indicated that the model had a sufficient convergence in the entire network (**S Figure 8**). Global I^2^ was employed to evaluate heterogeneity which was 0.4% for mortality. The model fit was estimated by comparing the posterior mean residual deviance with the number of data points, and the results were similar (**S Table 3**). Additionally, network meta-regression analysis revealed that study sample size, mean age, and gender did not significantly impact mortality (**S Figures 9–11**).

## Discussion

This is the first comprehensive Bayesian network meta-analysis of different antidiabetic agents for individuals with diabetes and COVID-19. When multiple treatment options are available, network meta-analysis with Bayesian statistics allows for robust mixed treatment comparison analysis. Furthermore, it enables combined direct and indirect comparisons of competing treatments while retaining the advantages of randomization (54). In this study, we assessed the association between various antidiabetic agents and mortality as well as the risk of severe disease in individuals with diabetes and COVID-19. This analysis included 42 studies and seven antidiabetic agents: metformin, DPP4i, sulfonylurea, TZDs, insulin, SGLT2i, and GLP1RA. The results revealed that metformin, DPP4i, SGLT2i, and GLP1RA treatment exhibited lower mortality than respective non-users, while insulin was linked to a higher mortality rate. Treatment with sulfonylurea and TZDs had no impact on mortality. Due to limited data, a meta-analysis of five antidiabetic agents (metformin, DPP4i, sulfonylurea, insulin, and SGLT2i) revealed no association with the risk of incidence of severe disease. Additionally, GLP1RA had the most significant protective effect against mortality among individuals with diabetes and COVID-19, followed by SGLT2i and metformin. Insulin was associated with the highest risk of mortality.

In this meta-analysis, metformin use was associated with decreased COVID-19 mortality, consistent with several previous studies (1, 3, 55). Moreover, statistical significance was observed between subgroups in the subgroup analyses, demonstrating that the overall results about metformin were stable and reliable. Inflammation has long been recognized as a critical factor in the development and severity of COVID-19. Severe COVID-19 is correlated with a hyperinflammatory state and cytokine storm, which can cause multi-organ damage (56). Metformin is the first-line drug in the treatment of diabetes. It has anti-inflammatory characteristics and has been shown to decrease circulating inflammatory biomarkers (2), which might explain why it is beneficial in diabetic patients with COVID-19. Furthermore, some scholars pointed out that metformin’s ability to reduce neutrophil counts and neutrophil extracellular traps might contribute to these protective mechanisms (57, 58).

In addition, we found that DPP4i use was associated with lower mortality, even when subgroup pooled adjusted data were included. A consistent conclusion has not been drawn in the previously published meta-analyses about DPP4i. The study (59) of Yang et al. concluded that DPP4i increased the mortality of patients with COVID-19 and diabetes, while Rakhmat’s study (60) concluded that DPP4i use was linked to decreased mortality. The other two meta-analyses (56, 61) suggested that DPP4i use was unrelated to mortality. The result of this contradictory evidence could be explained by the uncertain anti-inflammatory properties of DPP4i. In some studies, DPP4i treatment attenuated inflammasome activation and decreased human plasma levels of inflammatory biomarkers such as IL-6, IL-18, CRP, and so on (28, 62). However, some studies concluded that DPP4i did not affect human plasma levels of inflammatory biomarkers (56, 63). Additionally, the impact of DPP4i on active intact cytokines and chemokines can be paradoxical. By preventing inflammatory factors from being degraded enzymatically by DPP4, DPP4i may potentially enhance the activity of certain inflammatory networks (64), which might be the underlying reason for the high mortality observed by Yang et al. (59).

The pooled results of sulfonylurea from nine studies revealed no connection between sulfonylurea treatment and COVID-19 mortality. This finding contradicts a previous meta-analysis, in which five studies were included (3). Luk AOY et al. (21) reported a database that captured all COVID-19 patients in China Hong Kong, revealing that sulfonylurea use was associated with increased mortality. However, no statistical significance was observed in the remaining included studies. The specific potential mechanism remains unclear. TZDs is another glucose-lowering agent with the properties of modulating inflammatory and oxidative stress. However, some scholars pointed out that TZDs therapy was associated with weight gain, oedema, and heart failure, implying that it was unsuitable for COVID-19 patients (65). There was no meta-analysis about the effect of TZDs on COVID-19 so far. In this study, pooled results from five studies did not show any significant association between TZDs treatment and COVID-19 mortality as well as the risk of severe disease in diabetic patients.

In our meta-analysis, insulin administration was significantly associated with increased COVID-19 mortality, consistent with previous studies (1, 66). The underlying mechanism between the association of insulin use and COVID-19 remains unclear. Insulin exerted variable antioxidant and anti-inflammatory effects, including induction of nitric oxide production and inhibition of reactive oxygen species (67). Additionally, insulin therapy was proved reducing reactive oxygen species and macrophage infiltration into the liver and omentum in a diabetic rat model, resulting in a reduction in systematic inflammatory status (68). But in another sepsis model of diabetic rats, insulin showed a pro-inflammation ability in the lung (69). The lung was the main targeted organ affected by COVID-19. From a clinical perspective, some scholars worried that because insulin therapy was typically administered to patients with late-stage diabetes, it is difficult to exclude the negative effect of late-stage diabetes on the increased mortality. For instance, when insulin users were admitted to the hospital, they showed higher inflammatory markers, more comorbidities and lower lymphocyte counts than non-users (21). Based on the above, careful assessment of the benefits and adverse effects of insulin was required.

It is known that GLP1RA not only lowers glucose but also controls inflammation-induced lung injury through pulmonary protective effects and reduces major cardiovascular complications (70). Due to the prevalence of cardiovascular disease in diabetic patients, GLP1RA might be beneficial for glucose management in diabetic patients with COVID-19 (2). Moreover, GLP1RA works on GLP1 receptors primarily located on epithelial of the lung and immune cells. Rogliani et al. (71) reported that GLP1RA improved lung function in diabetic patients, while non-GLP1RA treatment or insulin treatment did not. Our meta-analysis corroborated the benefits of GLP1RA in managing COVID-19: lower mortality in GLP1RA users was observed compared to non-users.

SGLT2i is a new class of glucose-lowering agents, which has remarkable cardiorenal protective properties, especially in populations with atherosclerosis, heart failure, and reduced renal function (2, 72). Meanwhile, it has been shown to suppress systemic inflammation and fibrosis and alleviate organ damage (72, 73). The only RCT (53) included showed that patients treated with dapagliflozin had a lower but non-significant mortality. Our meta-analysis identified a significantly reduced mortality in SGLT2i users compared with non-users. Before this study, there have been no meta-analyses on the effect of SGLT2i on individuals with diabetes and COVID-19.

By conducting a Bayesian network meta-analysis on these seven antidiabetic agents to compare COVID-19 mortality between different antidiabetic agents, we discovered that GLP1RA had the most significant protective effect on COVID-19 mortality among individuals with diabetes, followed by SGLT2i and metformin. In contrast, insulin was associated with the highest risk of mortality. When metformin was used as a reference, GLP1RA and SGLT2i reduced mortality, whereas insulin increased mortality; no statistical significance was observed in other agents. Our Bayesian network meta-analysis demonstrated a good consistency of direct and indirect comparisons, indicating that the overall results were stable and reliable.

Although our comprehensive meta-analysis established a link between antidiabetic agents and the risk of COVID-19 clinical outcomes, our study has numerous limitations. First, most included studies were observational, including some confounding variables. We performed regression analysis on sample size, mean age, and male proportion. These results showed that these factors did not influence meta-analysis results. However, other confounding factors may remain. Second, in our pairwise meta-analysis, we did not perform an OR-to-RR effective size conversion and pooled OR and RR together. But we performed a subgroup analysis based on OR and RR. The results of subgroup analyses were consistent with those from the overall meta-analysis. Third, most of the included studies were observational, while only one RCT was included in the currently available literatures; thus, these results should be interpreted with caution.

## Conclusion

In conclusion, our thorough pairwise meta-analysis and Bayesian network meta-analysis revealed that using metformin, DPP4i, GLP1RA, and SGLT2i was high likely associated with decreased mortality in individuals with diabetes and COVID-19, while insulin treatment might contribute to the increased mortality. Using Sulfonylurea or TZDs had no statistically significant association with mortality. Furthermore, the meta-analysis found no association between the risk of severe disease and five antidiabetic agents (metformin, DPP4i, sulfonylurea, insulin, and SGLT2i). Comparative data between these antidiabetic agents revealed that GLP1RA probably had the most significant protective effect on COVID-19 mortality among individuals with diabetes, followed by SGLT2i and metformin. Insulin was correlated with the highest risk of mortality. More rigorous randomized controlled design investigations are required to further validate these findings.

## Data Availability Statement

The original contributions presented in the study are included in the article/**Supplementary Material**. Further inquiries can be directed to the corresponding author.

## Author Contributions

YC, XL, and SL: Conceptualization, methodology, data curation, investigation, and writing - original draft preparation. MA and MD: Supervision, writing - reviewing and editing, and funding acquisition. All authors contributed to the article and approved the submitted version.

## Funding 

The authors gratefully acknowledge the financial support by the science and technology Program of Science and Technology Commission Shanghai Municipality under Grant numbers 19441907000.

## Conflict of Interest

The authors declare that the research was conducted in the absence of any commercial or financial relationships that could be construed as a potential conflict of interest.

## Publisher’s Note

All claims expressed in this article are solely those of the authors and do not necessarily represent those of their affiliated organizations, or those of the publisher, the editors and the reviewers. Any product that may be evaluated in this article, or claim that may be made by its manufacturer, is not guaranteed or endorsed by the publisher.
