# Supplementary material for: The Association Between Antidiabetic Agents and Clinical Outcomes of COVID-19 Patients With Diabetes: A Bayesian Network Meta-Analysis
Source: Front Endocrinol (Lausanne). 2022 May 27;13:895458. doi: 10.3389/fendo.2022.895458 (PMC9186017; doi:10.3389/fendo.2022.895458)
Supplement: Supplementary file 1 [file DataSheet_1.docx]

**S Figures 1.** Subgroup analysis of different data types on mortality. (A) Metformin; (B) DPP4i; (C) Sulfonylurea; (D) Insulin.
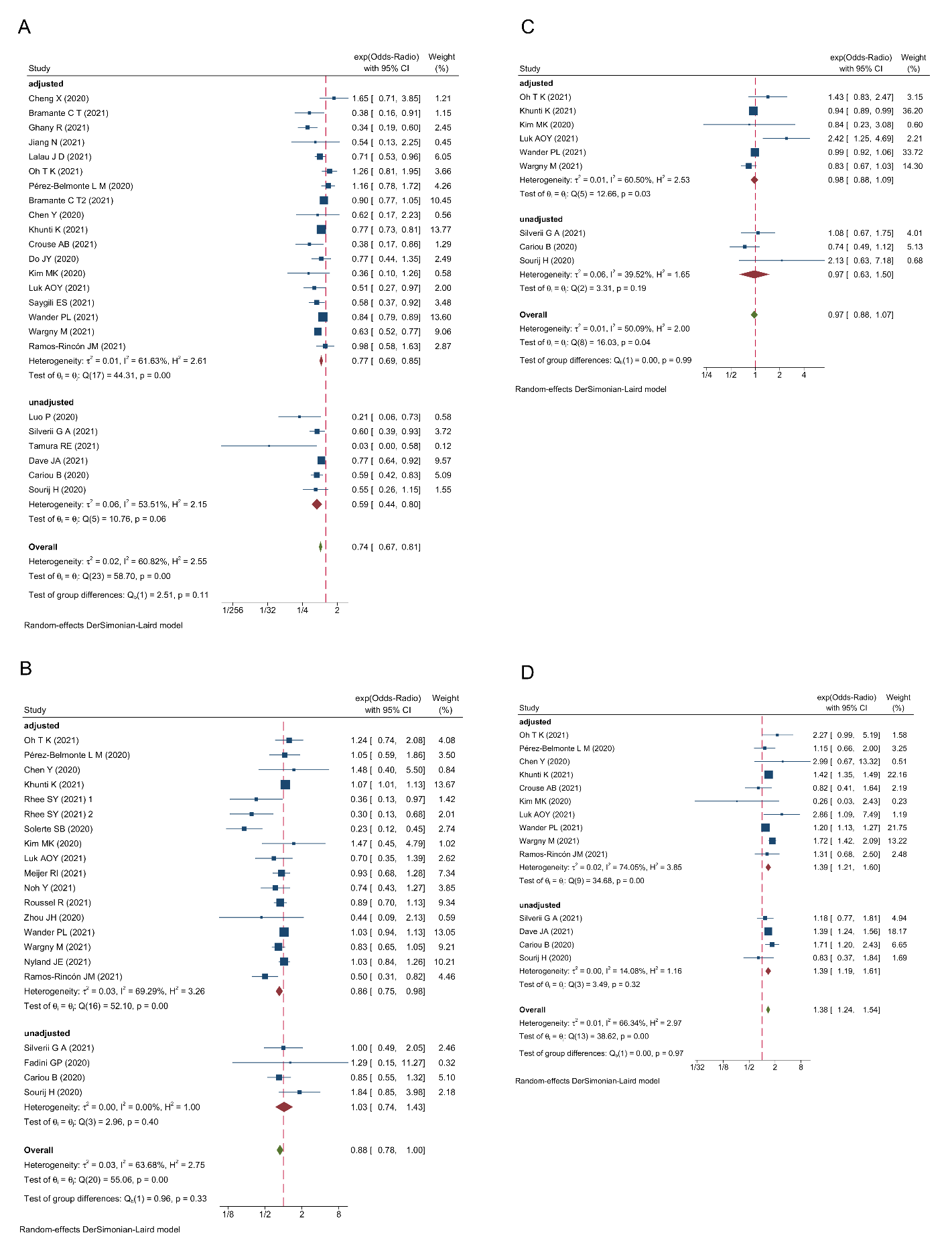


**S Figures 2.** Subgroup analysis of different effect size on mortality. (A) Metformin; (B) DPP4i; (C) Sulfonylurea; (D) Insulin.


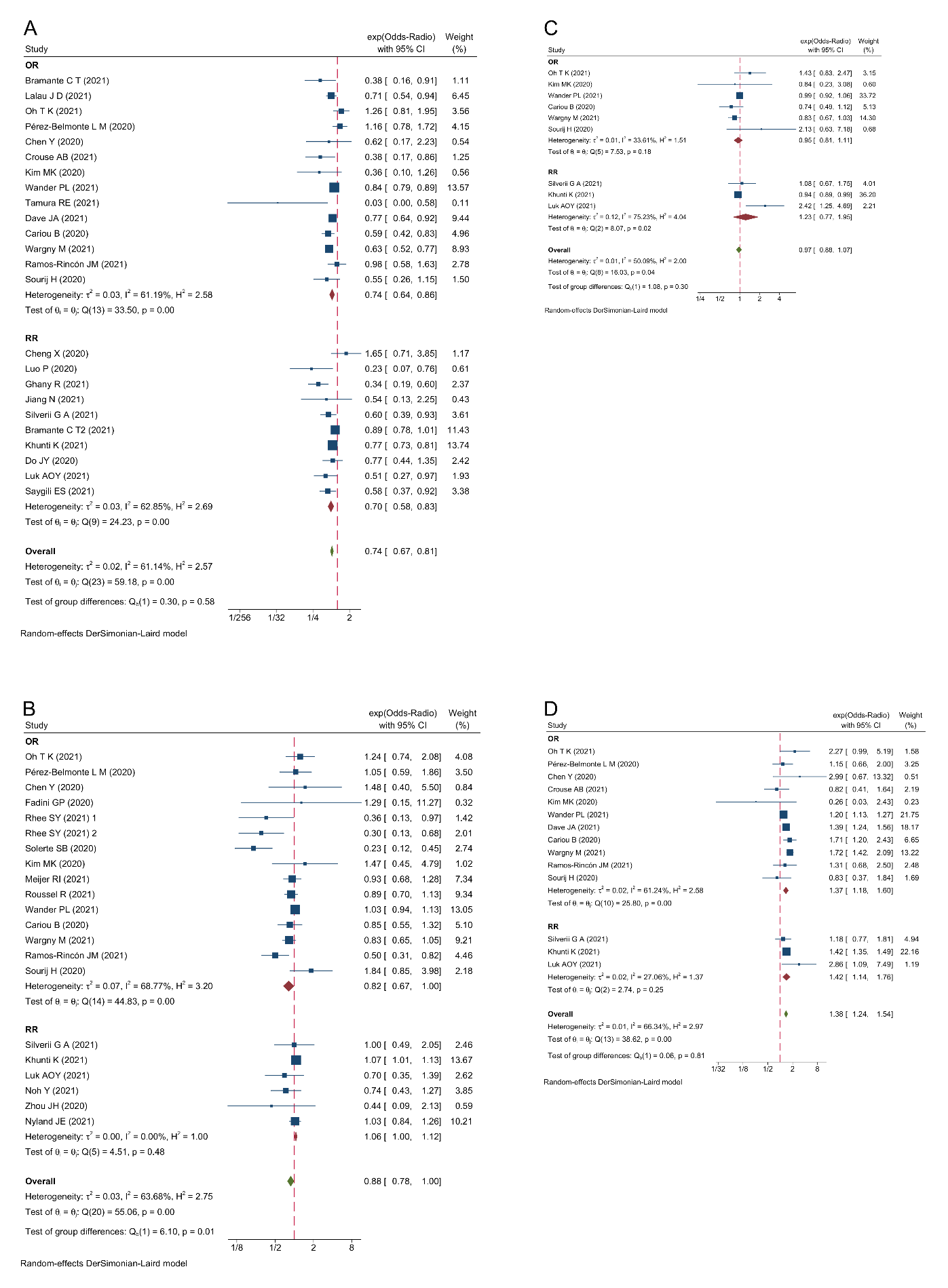


**S Figures 3.** Subgroup analysis of different diabetes types on mortality. (A) Metformin; (B) DPP4i; (C) Sulfonylurea; (D) Insulin.
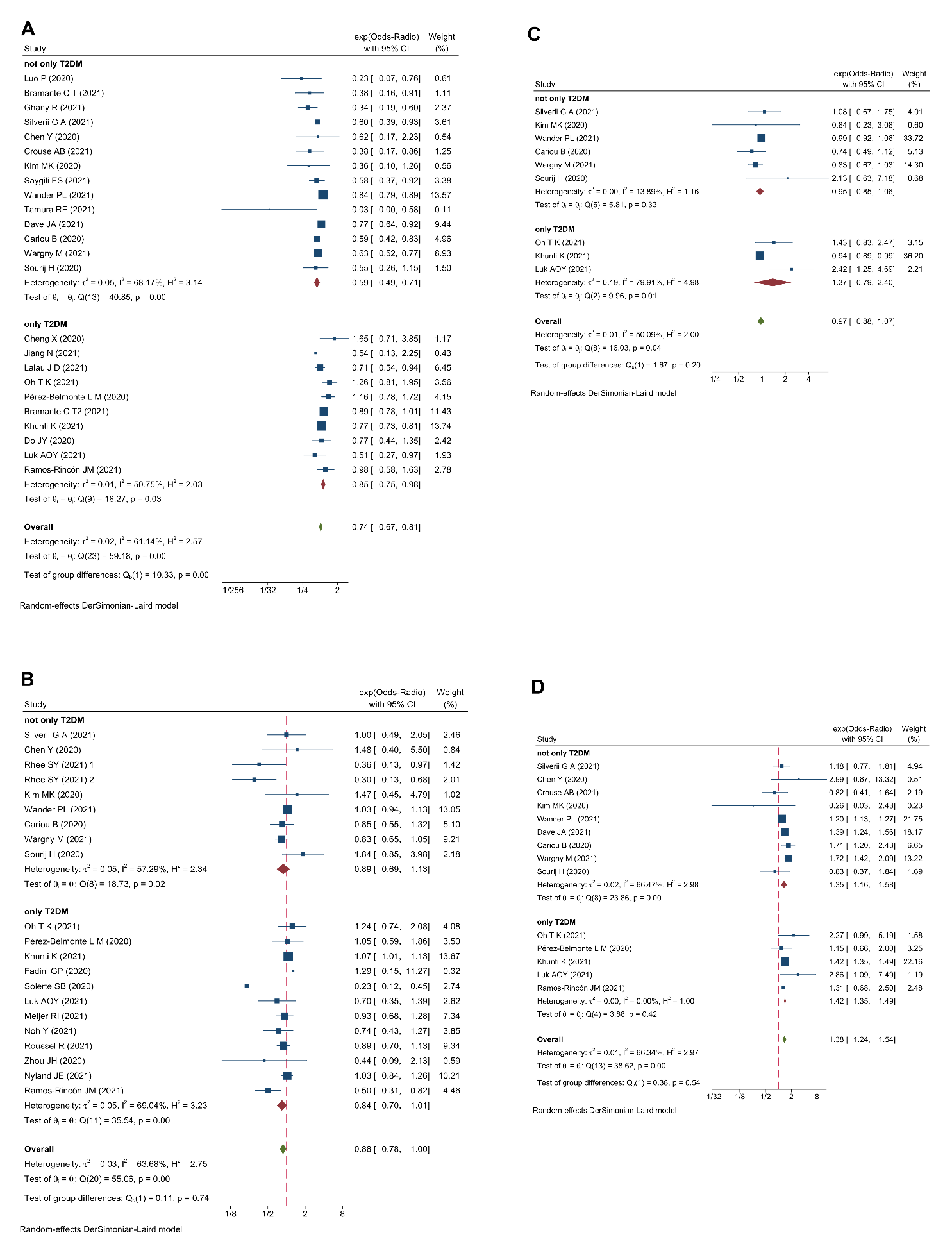


S Figures 4. Subgroup analysis of medication use timing on mortality. (A) Metformin; (B) DPP4i; (C) Sulfonylurea; (D) Insulin.
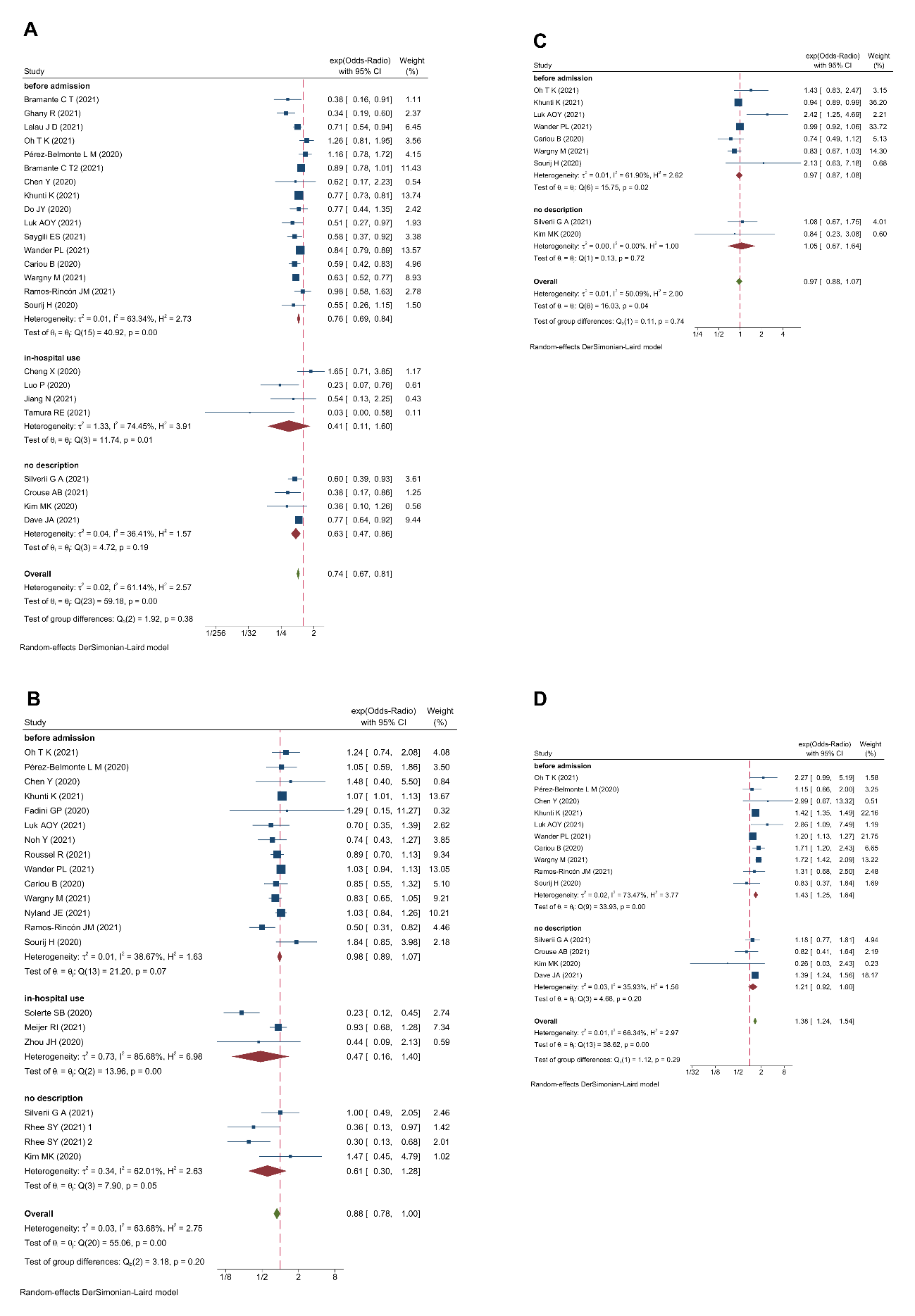


**S Figures 5.** Forest plot showed the association of antidiabetic agents use with the outcome of severe disease. (A) Metformin; (B) DPP4i; (C) Sulfonylurea; (D) Insulin; (E) SGLT2i.


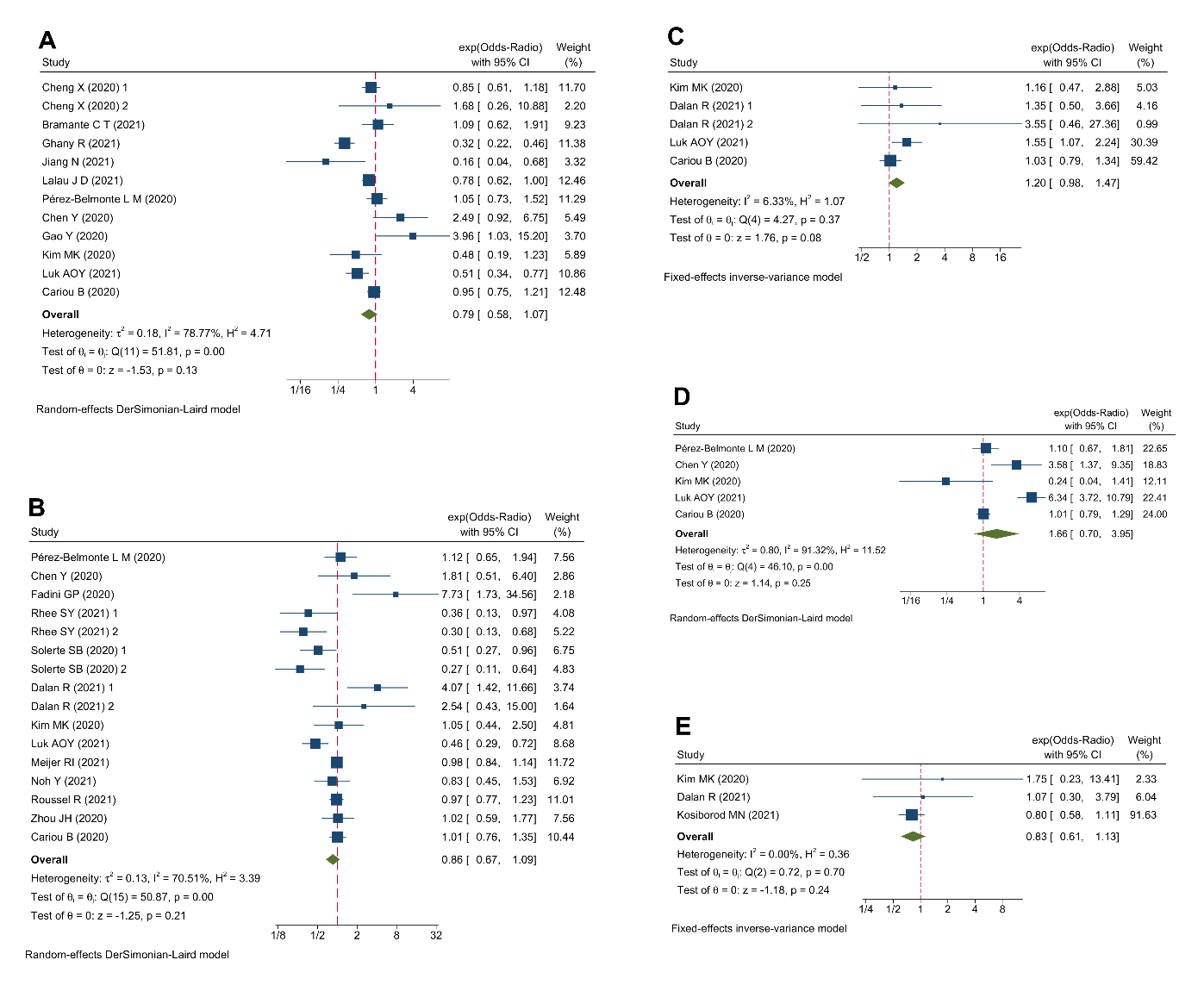


**S Figures 6.** The funnel plots for the mortality. (A) Metformin; (B) DPP4i; (C) Sulfonylurea; (D) TZDs; (E) Insulin; (F) SGLT2i; (G)GLP1RA.


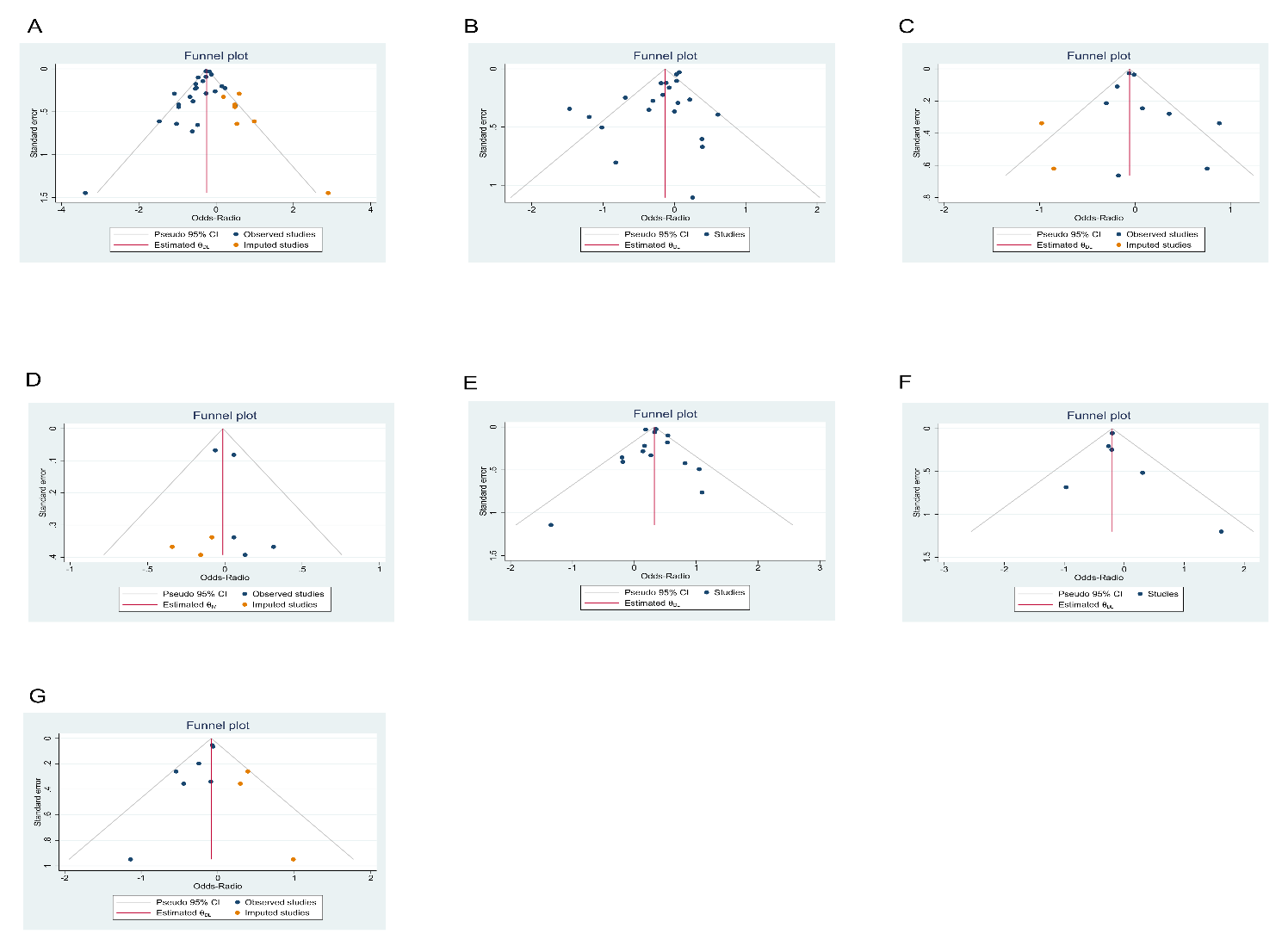


**S Figures 7.** Consistency analysis of direct and indirect comparison using node-splitting method.


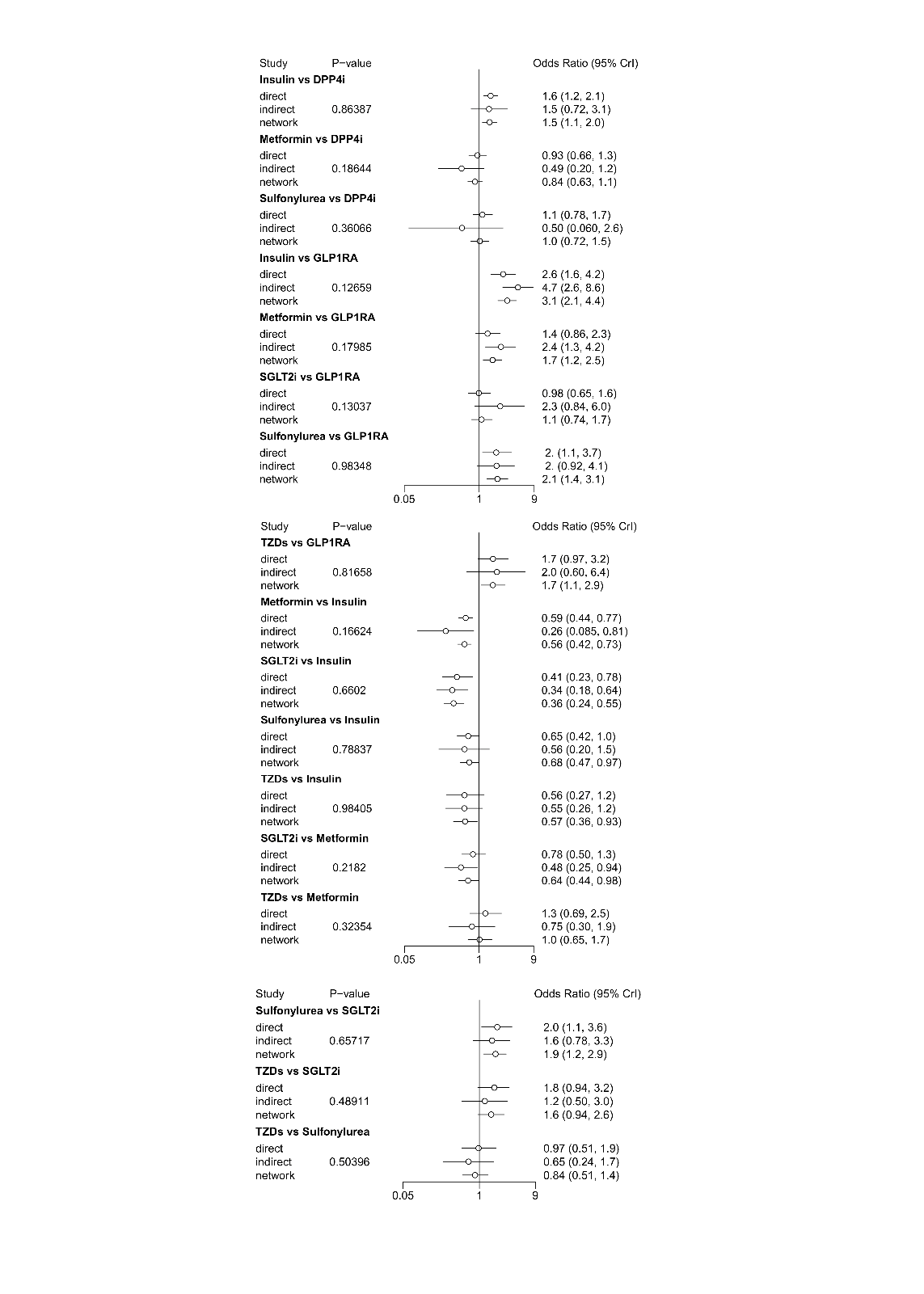


**S Figures 8.** The Brooks-Gelman-Rubin plots for the outcome of mortality in the Bayesian network meta-analysis.


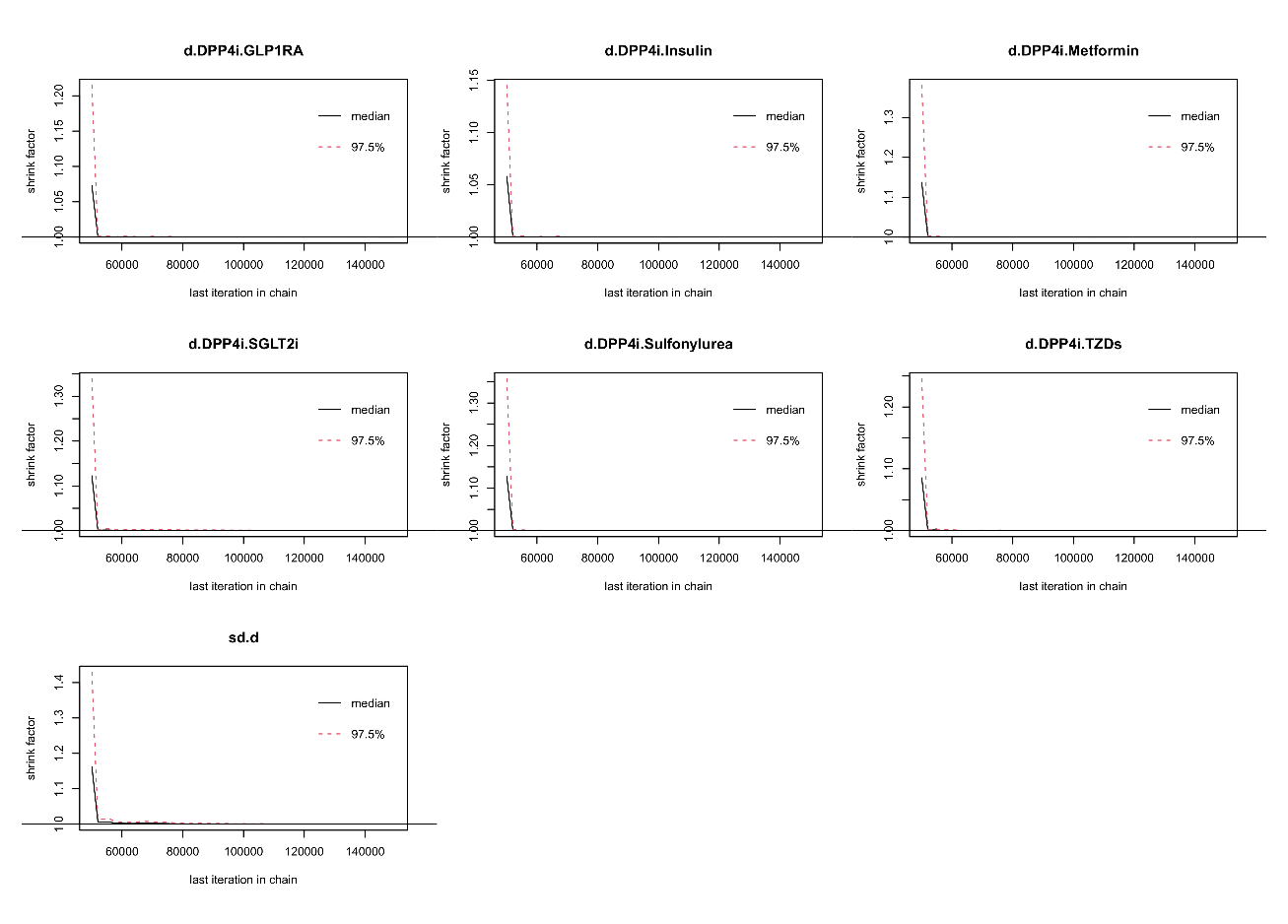


**S Figures 9.** Meta-regression analysis for the sample size between studies in the Bayesian network meta-analysis. (A) Insulin vs. DPP4i; (A) Insulin vs. GLP1RA; (A) Insulin vs. Metformin; (A) Insulin vs. SGLT2i; (A) Insulin vs. Sulfonylurea; (A) Insulin vs. TZDs.


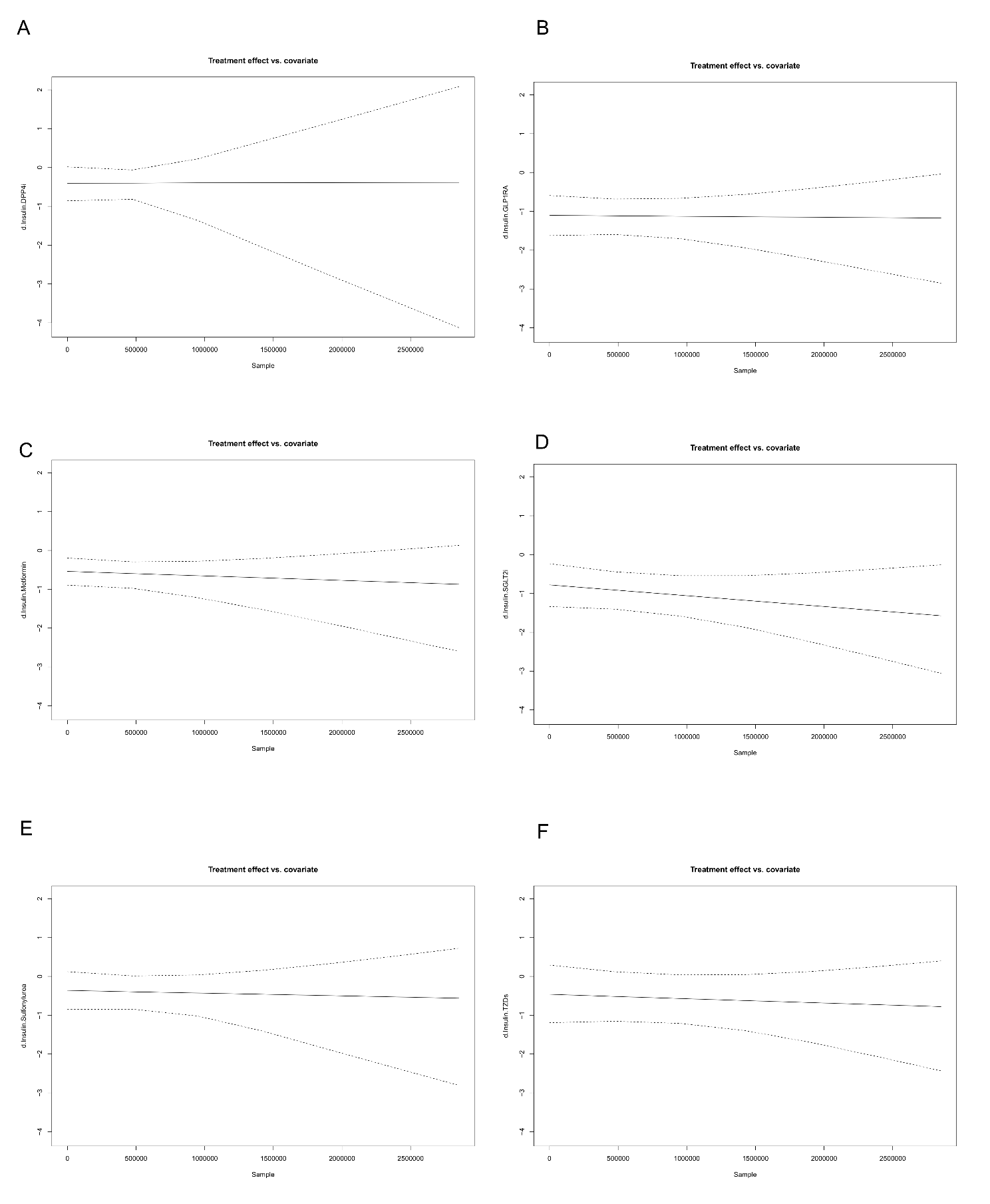


**S Figures 10.** Meta-regression analysis for the mean age between studies in the Bayesian network meta-analysis. (A) Insulin vs. DPP4i; (A) Insulin vs. GLP1RA; (A) Insulin vs. Metformin; (A) Insulin vs. SGLT2i; (A) Insulin vs. Sulfonylurea; (A) Insulin vs. TZDs.


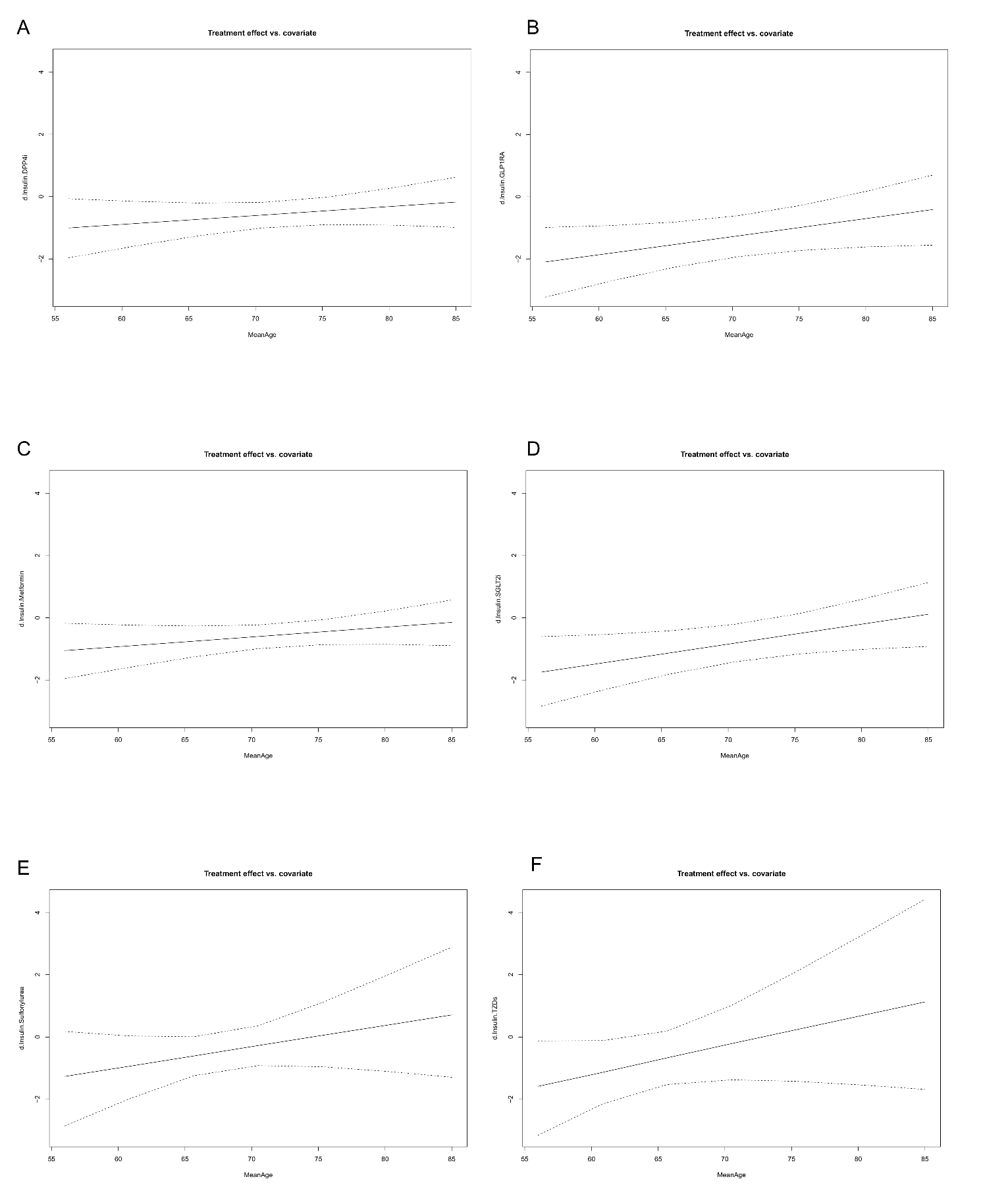


**S Figures 11.** Meta-regression analysis for the male proportion between studies in the Bayesian network meta-analysis. (A) Insulin vs. DPP4i; (A) Insulin vs. GLP1RA; (A) Insulin vs. Metformin; (A) Insulin vs. SGLT2i; (A) Insulin vs. Sulfonylurea; (A) Insulin vs. TZDs.


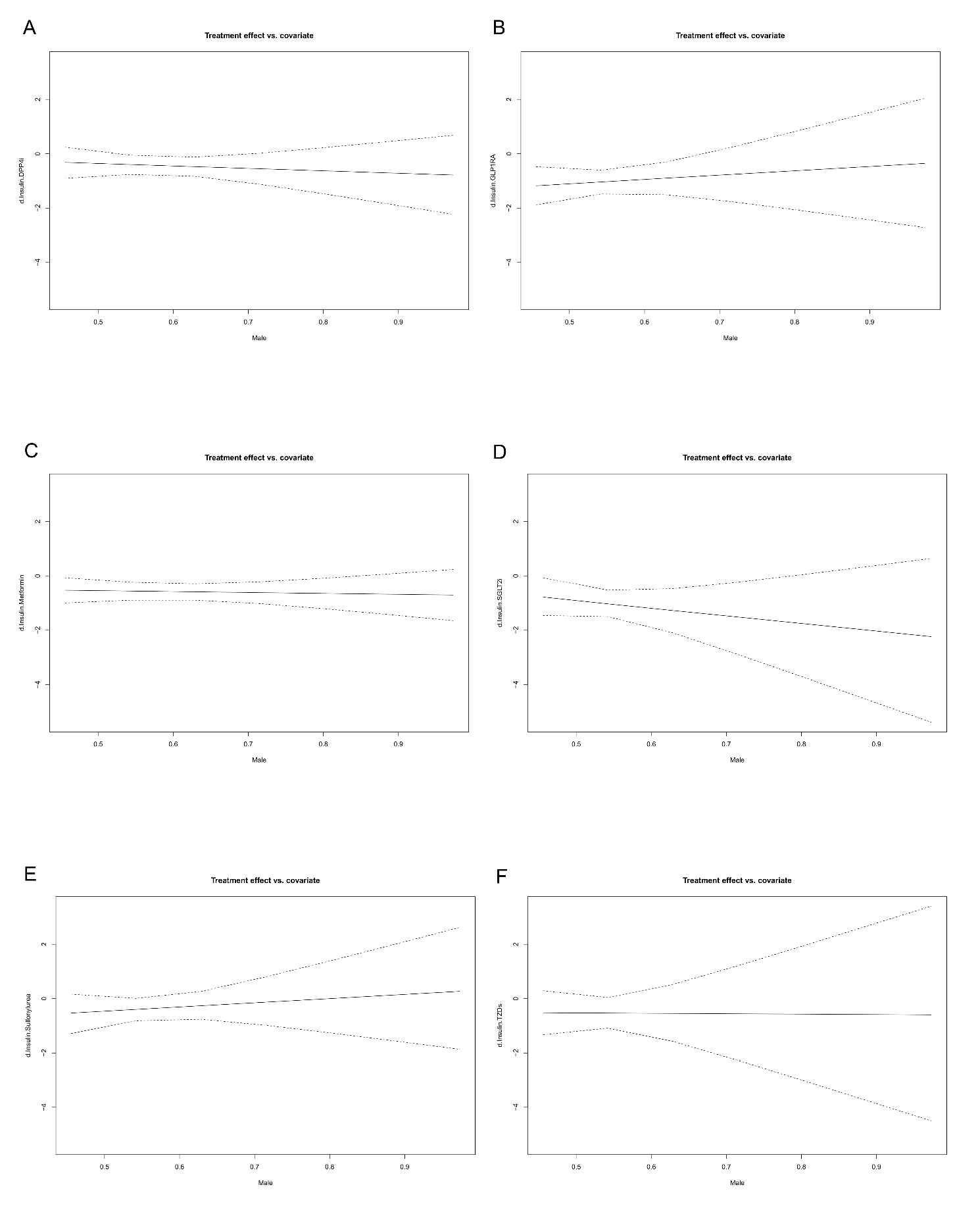


**S Table 1.** Search terms and strategies

| Metformin | Metformin OR Dimethylbiguanidine OR Dimethylguanylguanidine OR Glucophage |
| --- | --- |
| DPP4i | Dipeptidyl-Peptidase IV Inhibitors OR DPP-4 Inhibitors OR Sitagliptin OR Januvia OR Saxagliptin OR Onglyza OR Vildagliptin OR Galvus OR Alogliptin OR nesina OR Tiglitatin OR Linagliptin OR Tradjenta OR Trajenta |
| Sulfonylurea | Gliclazide OR Gliklazid OR Diamicron OR Diaglyk OR Glyade OR Diaikron OR Diabrezide OR Glimepiride OR Glimepiride OR Amaryl OR Amarel OR Gliquidone OR Glikvidon OR Glycvidon OR Glurenorm OR Beglynor OR Beglynora OR Glurenor OR Glipizide OR Glyburide OR Glypidizine OR Glidiazinamide OR Glydiazinamide OR Glucotrol OR Minidiab OR Mindiab OR Minodiab OR Melizide OR Ozidia OR Glupitel |
| TZDs | TZDs OR Thiazolidinediones OR Rosiglitazone OR Rosiglitazone Maleate OR Avandia OR Pioglitazone OR Pioglitazone Hydrochloride OR Actos |
| Glinides | Glinides OR Repaglinide OR Repa-glinide OR NovoNorm OR GlucoNorm OR Prandin OR Nateglinide OR Senaglinide OR Nate-Glinide OR Fastic OR Starsis OR Starlix OR Mitiglinide OR Miti-glinide |
| a-glycosidase inhibitors | a-glycosidase inhibitors OR Acarbose OR Glumida OR Glucobay OR Glucor OR Prandase OR Precose Voglibose OR Basen Miglitol OR Diastabol OR Plumarol OR Glyset |
| GLP1RA | Glucagon-Like Peptide-1 Receptor Agonists OR GLP-1 Receptor Agonists OR Exenatide OR Bydureon OR Exendin-4 OR Byetta OR Liraglutide OR Victoza OR Saxenda |
| SGLT2i | Sodium-glucose Co-transporter 2 Inhibitors OR SGLT-2 inhibitors OR Dapagliflozin OR Farxiga OR Forxiga OR Empagliflozin OR Jardiance OR Canagliflozin OR Invokana |
| Antidiabetic agents | Antidiabetic drugs OR Hypoglycemic agents OR Hypoglycemic drug |
| COVID-19 | COVID-19 OR COVID19 OR Coronavirus Disease 2019 OR 2019 novel coronavirus OR 2019-nCoV OR SARS-COV-2 |

**S Table 2.** Risk of bias assessment (Newcastle-Ottawa Quality Assessment Scale criteria).

| Study | Selection | Comparability | Outcome | Quality score |
| --- | --- | --- | --- | --- |
| Silverii, G. A (2021)[1] | **** | * | ** | 7 |
| Kahkoska, A. R (2021)[2] | **** | ** | ** | 8 |
| Ramos-Rincón, J. M (2021)[3] | **** | * | ** | 7 |
| Orioli, L (2021)[4] | **** | ** | ** | 8 |
| Israelsen, S. B (2021)[5] | **** | ** | ** | 8 |
| Sourij, H (2021)[6] | **** | ** | ** | 8 |
| Nyland, J. E (2021)[7] | **** | ** | ** | 8 |
| Cheng, X (2021)[8] | **** | ** | ** | 8 |
| Elibol, A (2021)[9] | **** | ** | ** | 8 |
| Luk, A. O. Y (2021)[10] | **** | ** | ** | 8 |
| Li, J (2021)[11] | **** | * | ** | 7 |
| Lally, M. A (2021)[12] | **** | ** | ** | 8 |
| Crouse, A. B (2020)[13] | **** | ** | ** | 8 |
| Pérez-Belmonte, L. M (2020)[14] | **** | ** | ** | 8 |
| Wargny, M (2021)[15] | **** | ** | ** | 8 |
| Khunti, K (2021)[16] | **** | ** | ** | 8 |
| Solerte, S. B (2020)[17] | **** | ** | ** | 8 |
| Meijer, R. I (2021)[18] | **** | ** | ** | 8 |
| Cheng X (2020)[19] | **** | ** | ** | 8 |
| Luo P (2020)[20] | **** | ** | ** | 8 |
| Bramante CT (2021)[21] | **** | ** | ** | 8 |
| Ghany R (2021)[22] | **** | * | ** | 7 |
| Jiang N (2021)[23] | **** | ** | ** | 8 |
| Lalau J D (2021)[24] | **** | ** | ** | 8 |
| Oh T K (2021)[25] | **** | ** | ** | 8 |
| Bramante CT2 (2021)[26] | **** | ** | ** | 8 |
| Chen Y (2020)[27] | **** | ** | ** | 8 |
| Do JY (2020)[28] | **** | ** | ** | 8 |
| Gao Y (2020)[29] | **** | * | ** | 7 |
| Kim MK (2020)[30] | **** | ** | ** | 8 |
| Saygili ES (2021)[31] | **** | ** | ** | 8 |
| Wander PL (2021)[32] | **** | * | ** | 7 |
| Tamura RE (2021)[33] | **** | ** | ** | 8 |
| Dave JA (2021)[34] | **** | * | ** | 7 |
| Cariou B (2020)[35] | **** | ** | ** | 8 |
| Fadini GP (2020)[36] | **** | * | ** | 7 |
| Rhee SY (2021)[37] | **** | ** | ** | 8 |
| Dalan R (2021)[38] | **** | ** | ** | 8 |
| Noh Y (2021)[39] | **** | ** | ** | 8 |
| Roussel R (2021)[40] | **** | ** | ** | 8 |
| Zhou JH (2020)[41] | **** | ** | ** | 8 |
| Kosiborod MN (2021)[42] | **** | ** | *** | 9 |

**S Table 3.** Assessment of model fit. If the difference of DIC value in two modes is within 5, it means that the data is consistent.

| **Outcome** | **Consistency model** | | | | | **Inconsistency model** | | | | |
| --- | --- | --- | --- | --- | --- | --- | --- | --- | --- | --- |
|  | Data points | Residual deviance | Ratio | I^2^ | DIC | Data points | Residual deviance | Ratio | I^2^ | DIC |
| Mortality | 74 | 73.33 | 0.99 | 0.5% | 123.05 | 74 | 71.06 | 0.96 | 0% | 123.36 |

Abbreviation: DIC, deviance information criterion.

[1] Silverii GA, Monami M, Cernigliaro A, Vigneri E, Guarnotta V, Scondotto S, et al. Are diabetes and its medications risk factors for the development of COVID-19? Data from a population-based study in Sicily. Nutrition, metabolism, and cardiovascular diseases : NMCD. 2021;31:396-8.

[2] Kahkoska AR, Abrahamsen TJ, Alexander GC, Bennett TD, Chute CG, Haendel MA, et al. Association Between Glucagon-Like Peptide 1 Receptor Agonist and Sodium-Glucose Cotransporter 2 Inhibitor Use and COVID-19 Outcomes. Diabetes care. 2021;44:1564-72.

[3] Ramos-Rincón JM, Pérez-Belmonte LM, Carrasco-Sánchez FJ, Jansen-Chaparro S, De-Sousa-Baena M, Bueno-Fonseca J, et al. Cardiometabolic Therapy and Mortality in Very Old Patients With Diabetes Hospitalized due to COVID-19. The journals of gerontology Series A, Biological sciences and medical sciences. 2021;76:e102-e9.

[4] Orioli L, Servais T, Belkhir L, Laterre P-F, Thissen J-P, Vandeleene B, et al. Clinical characteristics and short-term prognosis of in-patients with diabetes and COVID-19: A retrospective study from an academic center in Belgium. Diabetes & metabolic syndrome. 2021;15:149-57.

[5] Israelsen SB, Pottegård A, Sandholdt H, Madsbad S, Thomsen RW, Benfield T. Comparable COVID-19 outcomes with current use of GLP-1 receptor agonists, DPP-4 inhibitors or SGLT-2 inhibitors among patients with diabetes who tested positive for SARS-CoV-2. Diabetes, obesity & metabolism. 2021;23:1397-401.

[6] Sourij H, Aziz F, Bräuer A, Ciardi C, Clodi M, Fasching P, et al. COVID-19 fatality prediction in people with diabetes and prediabetes using a simple score upon hospital admission. Diabetes, obesity & metabolism. 2021;23:589-98.

[7] Nyland JE, Raja-Khan NT, Bettermann K, Haouzi PA, Leslie DL, Kraschnewski JL, et al. Diabetes, Drug Treatment and Mortality in COVID-19: A Multinational Retrospective Cohort Study. Diabetes. 2021.

[8] Cheng X, Xin S, Chen Y, Li L, Chen W, Li W, et al. Effects of metformin, insulin on COVID-19 patients with pre-existed type 2 diabetes: A multicentral retrospective study. Life Sci. 2021;275:119371.

[9] Elibol A, Eren D, Erdoğan MD, Elmaağaç M, Dizdar OS, Çelik İ, et al. Factors influencing on development of COVID-19 pneumonia and association with oral anti-diabetic drugs in hospitalized patients with diabetes mellitus. Primary care diabetes. 2021;15:806-12.

[10] Luk AOY, Yip TCF, Zhang X, Kong APS, Wong VW, Ma RCW, et al. Glucose-lowering drugs and outcome from COVID-19 among patients with type 2 diabetes mellitus: a population-wide analysis in Hong Kong. BMJ Open. 2021;11:e052310.

[11] Li J, Wei Q, McCowen KC, Xiong W, Liu J, Jiang W, et al. Inpatient use of metformin and acarbose is associated with reduced mortality of COVID-19 patients with type 2 diabetes mellitus. Endocrinology, diabetes & metabolism. 2021:e00301.

[12] Lally MA, Tsoukas P, Halladay CW, O'Neill E, Gravenstein S, Rudolph JL. Metformin is Associated with Decreased 30-Day Mortality Among Nursing Home Residents Infected with SARS-CoV2. J Am Med Dir Assoc. 2021;22:193-8.

[13] Crouse AB, Grimes T, Li P, Might M, Ovalle F, Shalev A. Metformin Use Is Associated With Reduced Mortality in a Diverse Population With COVID-19 and Diabetes. Front Endocrinol (Lausanne). 2020;11:600439.

[14] Pérez-Belmonte LM, Torres-Peña JD, López-Carmona MD, Ayala-Gutiérrez MM, Fuentes-Jiménez F, Huerta LJ, et al. Mortality and other adverse outcomes in patients with type 2 diabetes mellitus admitted for COVID-19 in association with glucose-lowering drugs: a nationwide cohort study. BMC Med. 2020;18:359.

[15] Wargny M, Potier L, Gourdy P, Pichelin M, Amadou C, Benhamou PY, et al. Predictors of hospital discharge and mortality in patients with diabetes and COVID-19: updated results from the nationwide CORONADO study. Diabetologia. 2021;64:778-94.

[16] Khunti K, Knighton P, Zaccardi F, Bakhai C, Barron E, Holman N, et al. Prescription of glucose-lowering therapies and risk of COVID-19 mortality in people with type 2 diabetes: a nationwide observational study in England. The lancet Diabetes & endocrinology. 2021;9:293-303.

[17] Solerte SB, D'Addio F, Trevisan R, Lovati E, Rossi A, Pastore I, et al. Sitagliptin Treatment at the Time of Hospitalization Was Associated With Reduced Mortality in Patients With Type 2 Diabetes and COVID-19: A Multicenter, Case-Control, Retrospective, Observational Study. Diabetes Care. 2020;43:2999-3006.

[18] Meijer RI, Hoekstra T, van den Oever NCG, Simsek S, van den Bergh JP, Douma RA, et al. Treatment with a DPP-4 inhibitor at time of hospital admission for COVID-19 is not associated with improved clinical outcomes: data from the COVID-PREDICT cohort study in The Netherlands. Journal of diabetes and metabolic disorders. 2021:1-6.

[19] Cheng X, Liu YM, Li H, Zhang X, Lei F, Qin JJ, et al. Metformin Is Associated with Higher Incidence of Acidosis, but Not Mortality, in Individuals with COVID-19 and Pre-existing Type 2 Diabetes. Cell Metab. 2020;32:537-47 e3.

[20] Luo P, Qiu L, Liu Y, Liu XL, Zheng JL, Xue HY, et al. Metformin Treatment Was Associated with Decreased Mortality in COVID-19 Patients with Diabetes in a Retrospective Analysis. The American journal of tropical medicine and hygiene. 2020;103:69-72.

[21] Bramante CT, Buse J, Tamaritz L, Palacio A, Cohen K, Vojta D, et al. Outpatient metformin use is associated with reduced severity of COVID-19 disease in adults with overweight or obesity. J Med Virol. 2021;93:4273-9.

[22] Ghany R, Palacio A, Dawkins E, Chen G, McCarter D, Forbes E, et al. Metformin is associated with lower hospitalizations, mortality and severe coronavirus infection among elderly medicare minority patients in 8 states in USA. Diabetes & metabolic syndrome. 2021;15:513-8.

[23] Jiang N, Chen Z, Liu L, Yin X, Yang H, Tan X, et al. Association of metformin with mortality or ARDS in patients with COVID-19 and type 2 diabetes: A retrospective cohort study. Diabetes Res Clin Pract. 2021;173:108619.

[24] Lalau JD, Al-Salameh A, Hadjadj S, Goronflot T, Wiernsperger N, Pichelin M, et al. Metformin use is associated with a reduced risk of mortality in patients with diabetes hospitalised for COVID-19. Diabetes & metabolism. 2021;47:101216.

[25] Oh TK, Song IA. Metformin use and risk of COVID-19 among patients with type II diabetes mellitus: an NHIS-COVID-19 database cohort study. Acta Diabetol. 2021;58:771-8.

[26] Bramante CT, Ingraham NE, Murray TA, Marmor S, Hovertsen S, Gronski J, et al. Metformin and risk of mortality in patients hospitalised with COVID-19: a retrospective cohort analysis. The Lancet Healthy longevity. 2021;2:e34-e41.

[27] Chen Y, Yang D, Cheng B, Chen J, Peng A, Yang C, et al. Clinical Characteristics and Outcomes of Patients With Diabetes and COVID-19 in Association With Glucose-Lowering Medication. Diabetes Care. 2020;43:1399-407.

[28] Do JY, Kim SW, Park JW, Cho KH, Kang SH. Is there an association between metformin use and clinical outcomes in diabetes patients with COVID-19? Diabetes & metabolism. 2021;47:101208.

[29] Gao Y, Liu T, Zhong W, Liu R, Zhou H, Huang W, et al. Risk of Metformin in Patients With Type 2 Diabetes With COVID-19: A Preliminary Retrospective Report. Clin Transl Sci. 2020;13:1055-9.

[30] Kim MK, Jeon JH, Kim SW, Moon JS, Cho NH, Han E, et al. The Clinical Characteristics and Outcomes of Patients with Moderate-to-Severe Coronavirus Disease 2019 Infection and Diabetes in Daegu, South Korea. Diabetes & metabolism journal. 2020;44:602-13.

[31] Saygili ES, Karakiliç E, Mert E, Şener A, Mirci A. Preadmission usage of metformin and mortality in COVID-19 patients including the post-discharge period. Irish journal of medical science. 2021:1-7.

[32] Wander PL, Lowy E, Beste LA, Tulloch-Palomino L, Korpak A, Peterson AC, et al. Prior Glucose-Lowering Medication Use and 30-Day Outcomes Among 64,892 Veterans With Diabetes and COVID-19. Diabetes Care. 2021;44:2708-13.

[33] Tamura RE, Said SM, de Freitas LM, Rubio IGS. Outcome and death risk of diabetes patients with Covid-19 receiving pre-hospital and in-hospital metformin therapies. Diabetology & metabolic syndrome. 2021;13:76.

[34] Dave JA, Tamuhla T, Tiffin N, Levitt NS, Ross IL, Toet W, et al. Risk factors for COVID-19 hospitalisation and death in people living with diabetes: A virtual cohort study from the Western Cape Province, South Africa. Diabetes Res Clin Pract. 2021;177:108925.

[35] Cariou B, Hadjadj S, Wargny M, Pichelin M, Al-Salameh A, Allix I, et al. Phenotypic characteristics and prognosis of inpatients with COVID-19 and diabetes: the CORONADO study. Diabetologia. 2020;63:1500-15.

[36] Fadini GP, Morieri ML, Longato E, Bonora BM, Pinelli S, Selmin E, et al. Exposure to dipeptidyl-peptidase-4 inhibitors and COVID-19 among people with type 2 diabetes: A case-control study. Diabetes, obesity & metabolism. 2020;22:1946-50.

[37] Rhee SY, Lee J, Nam H, Kyoung DS, Shin DW, Kim DJ. Effects of a DPP-4 Inhibitor and RAS Blockade on Clinical Outcomes of Patients with Diabetes and COVID-19. Diabetes & metabolism journal. 2021;45:251-9.

[38] Dalan R, Ang LW, Tan WYT, Fong SW, Tay WC, Chan YH, et al. The association of hypertension and diabetes pharmacotherapy with COVID-19 severity and immune signatures: an observational study. Eur Heart J Cardiovasc Pharmacother. 2021;7:e48-e51.

[39] Noh Y, Oh IS, Jeong HE, Filion KB, Yu OHY, Shin JY. Association Between DPP-4 Inhibitors and COVID-19-Related Outcomes Among Patients With Type 2 Diabetes. Diabetes Care. 2021;44:e64-e6.

[40] Roussel R, Darmon P, Pichelin M, Goronflot T, Abouleka Y, Ait Bachir L, et al. Use of dipeptidyl peptidase-4 inhibitors and prognosis of COVID-19 in hospitalized patients with type 2 diabetes: A propensity score analysis from the CORONADO study. Diabetes, obesity & metabolism. 2021;23:1162-72.

[41] Zhou JH, Wu B, Wang WX, Lei F, Cheng X, Qin JJ, et al. No significant association between dipeptidyl peptidase-4 inhibitors and adverse outcomes of COVID-19. World J Clin Cases. 2020;8:5576-88.

[42] Kosiborod MN, Esterline R, Furtado RHM, Oscarsson J, Gasparyan SB, Koch GG, et al. Dapagliflozin in patients with cardiometabolic risk factors hospitalised with COVID-19 (DARE-19): a randomised, double-blind, placebo-controlled, phase 3 trial. The lancet Diabetes & endocrinology. 2021;9:586-94.
